# Isolation of antigen-specific, disulphide-rich knob domain peptides from bovine antibodies

**DOI:** 10.1371/journal.pbio.3000821

**Published:** 2020-09-04

**Authors:** Alex Macpherson, Anthony Scott-Tucker, Anastasios Spiliotopoulos, Catherine Simpson, Justin Staniforth, Adam Hold, James Snowden, Leah Manning, Jean van den Elsen, Alastair D. G. Lawson

**Affiliations:** 1 UCB, Slough, United Kingdom; 2 Department of Biology and Biochemistry, University of Bath, Bath, United Kingdom; Scripps Research Institute, UNITED STATES

## Abstract

As a novel alternative to established surface display or combinatorial chemistry approaches for the discovery of therapeutic peptides, we present a method for the isolation of small, cysteine-rich domains from bovine antibody ultralong complementarity-determining regions (CDRs). We show for the first time that isolated bovine antibody knob domains can function as autonomous entities by binding antigen outside the confines of the antibody scaffold. This yields antibody fragments so small as to be considered peptides, each stabilised by an intricate, bespoke arrangement of disulphide bonds. For drug discovery, cow immunisations harness the immune system to generate knob domains with affinities in the picomolar to low nanomolar range, orders of magnitude higher than unoptimized peptides from naïve library screening. Using this approach, knob domain peptides that tightly bound Complement component C5 were obtained, at scale, using conventional antibody discovery and peptide purification techniques.

## Abstract 2

This study describes a method for the isolation of knob domains (a disulfide-rich domain found in the ultra-long CDRH3 of a subset of bovine antibodies) to create a uniquely small antibody fragment. With a molecular weight 3-6 KDa, the knob domain fragment is so small as to be considered a peptide. This approach uniquely harnesses the bovine immune system to affinity maturate peptides in vivo.

## Introduction

**Fig 1:**
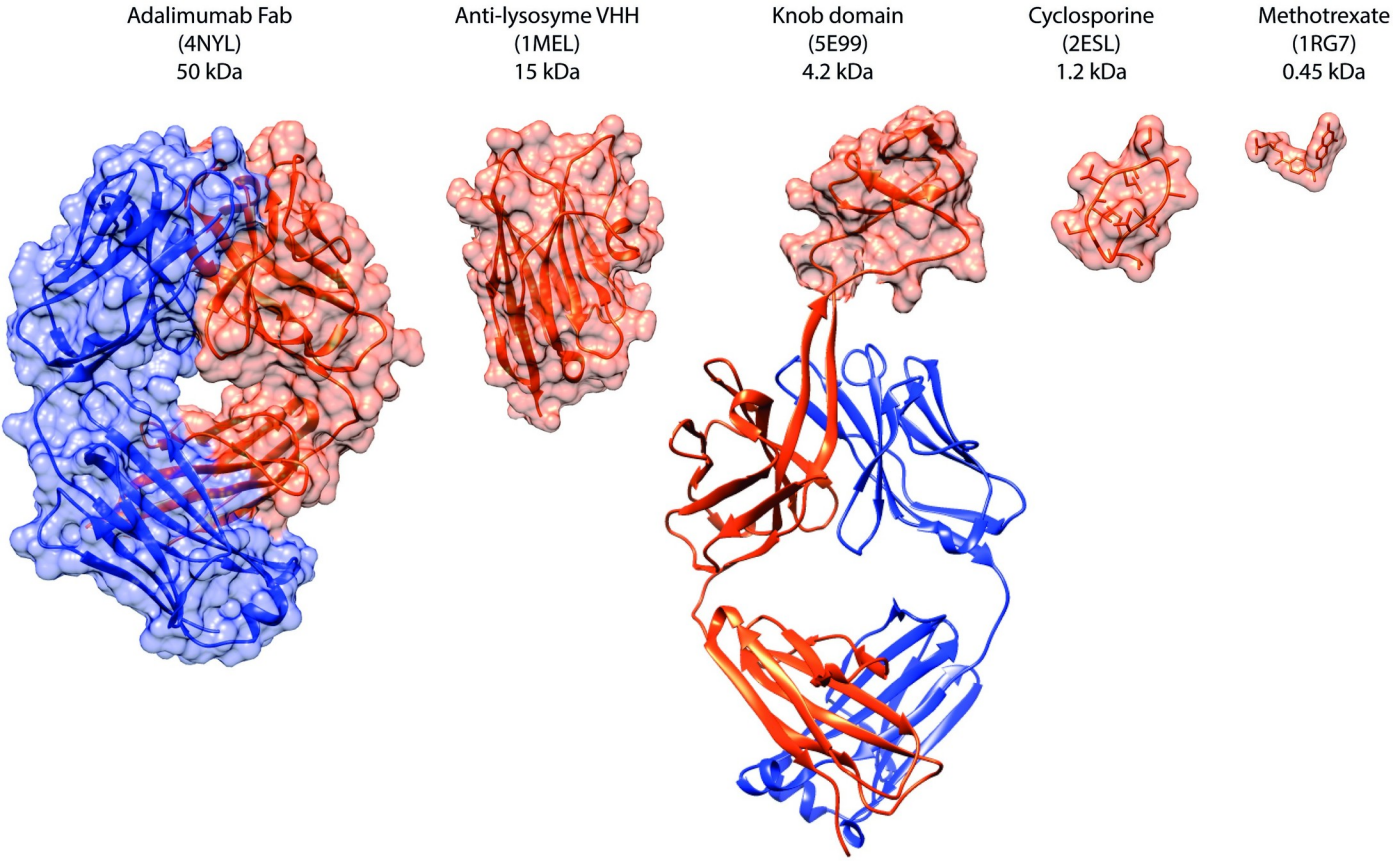
Therapeutic molecules by size. Molecular size comparison of therapeutic molecules, ranging from the 50 kDa Adalimumab Fab fragment to the 450 Da small-molecule methotrexate. A VHH antibody fragment (MW 15 kDa), derived from the heavy-chain–only subset of the camelid repertoire, is shown alongside the knob domain peptide of a bovine ultralong CDRH3 (MW 3–5 kDa), that bridges the gap between the antibody fragments and cyclic peptides, such as cyclosporin (MW 1.2 kDa). Ribbon diagrams and molecular surface representations were produced using UCSF Chimera [13]. PDB accession codes for the molecular structures used in this figure are indicated. CDRH3, heavy-chain complementarity-determining region 3; MW, molecular weight; PDB, Protein Data Bank; VHH, variable regions of camelid heavy-chain antibodies.

To date, the smallest autonomous, naturally occurring, functional antibody domains reported have been the variable regions of camelid heavy-chain antibodies (VHH) [1] and the variable regions of the immunoglobulin new antigen receptor (VNAR), derived from sharks [2], resulting in heavy-chain variable region fragments, of some 12–15 kDa [3, 4]. In 1997, the first report of bovine antibodies featuring an ultralong heavy-chain complementarity-determining region 3 (CDRH3) was published [5], with subsequent crystal structures revealing a conserved anti-parallel β-ribbon ‘stalk’ presenting a disulphide-stabilised ‘knob’ domain of 3–6 KDa [6]. The knob domain, shown in Fig 1, comprises several small loops stapled by 2 to 5 disulphide bonds and is held some 25–45 Å clear of the surface of the other complementarity-determining region (CDR) loops [6–8]. Mutation studies with whole IgG have shown that binding can occur predominantly through the knob domain with limited input from neighbouring CDRs [6], but the surrounding architecture of the antibody scaffold is still thought to play a critical supporting role [6–10]. Recently, the first co-crystal structures of an ultralong CDRH3 Fab in complex with antigen—in this case, an anti-HIV broadly neutralising antibody [11] bound to a soluble portion of the envelope glycoprotein GP120—found that antigen recognition was contained entirely within the ultralong CDRH3 [12]. The study notes a nuanced role for the stalk, with alanine mutations in this region maintaining binding affinity but attenuating neutralisation efficiency [12]. In this paper, we show that, when isolated from the parent antibody infrastructure and the stalk, the bovine knob domain itself may be able to bind to antigen with high affinity and so can function as an autonomous entity.

Bovine ultralong CDRH3 arise due to the limited repertoire of antibody gene segments in domesticated cattle. Within their antibody heavy chain repertoire, cows have approximately 10× highly homologous V_H_, 10× D_H_, and just 4× J_H_ segments, giving a theoretical heavy chain diversity of only approximately 4,000 [14]; by contrast, humans have 55× V_H_, 23× D_H_, and 6× J_H_ segments, giving a theoretical diversity of up to 7,590 [15]. Cows exploit other mechanisms for generating antibody diversity. In addition to relying heavily on somatic hypermutation (SHM), bovines have evolved a longer CDRH3, even amongst the non-ultralong repertoire, maximising the value from rearrangement of their limited immune gene subset. Bovines have also evolved a mechanism for overcoming the conformational entropy penalty associated with long loops by using disulphide bonds to staple the CDRH3 in the biologically active conformer.

To facilitate disulphide bond formation, the introduction of cysteines is achieved through priming of a germline D-gene segment, immunoglobulin heavy chain diversity region 8–2 (IGHD8-2), which exclusively encodes the central portion of ultralong CDRH3. The germline peptide contains 4 cysteines, but glycine, tyrosine, and serine residues are also prominent, each of which can be mutated to cysteine by a single nucleotide change during SHM [16]. Interestingly, codon use within IGHD8-2 seems to favour glycine and tyrosine codons that are otherwise infrequently used in the bovine genome but are closer to the TGT/TGC encoding cysteine, hardwiring for this specific mutation [10]. Extended CDRH3 length and complexity has also been described in *Ornithorhynchus anatinus*, the Australian duck-billed platypus, but stabilisation occurs predominantly through inter-CDR disulphide bonds, again, in response to a paucity of V-gene segments [17]. Currently, complex stabilisation of CDRH3 knob domains with intra-CDR disulphide bonds appears unique to cattle.

In order to enable the generation of isolated knob domains, we performed bovine antibody discovery against Complement component C5, a 188-kDa humoral protein of the complement cascade, as the test antigen. C5 is the target of the Food and Drug Administration (FDA)-approved monoclonal antibody eculizumab in diseases such as paroxysmal nocturnal haemoglobinuria (PNH) [18, 19] and atypical haemolytic uremic syndrome (aHUS) [20] and remains an enduring focus of drug discovery efforts to prevent complement-induced autoinflammation, with a number of second-generation therapeutics in clinical and preclinical development [21]. Fig 1 shows how isolated knob domains would effectively bridge a molecular weight gap between camelid-derived VHH antibody domains and chemical macrocycles, with potential for therapeutic utility.

**Fig 2:**
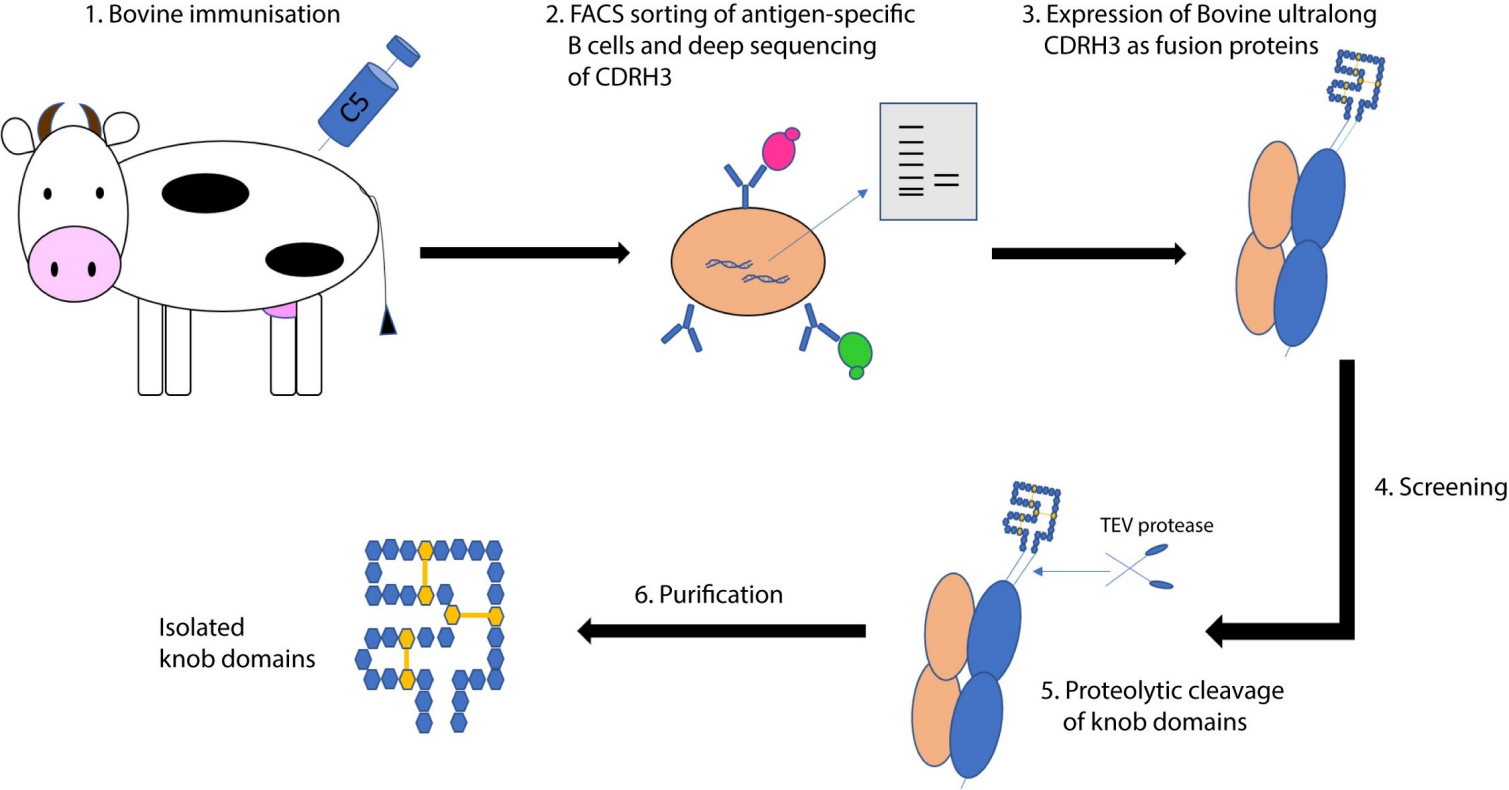
Schematic representation of the method for the isolation of knob domain peptides. Immunisation of cattle with human C5 is used to elicit an immune response (step 1). An antigen-specific response is isolated through FACS of immune cells, using 2 populations of fluorescently labelled C5 (step 2). Cells that are double positive for C5 are sorted into a polyclonal mixture. After RT-PCR, PCR primers specific to CDRH3 are used to create a CDRH3 library. Ultralong CDRH3 are identified using deep sequencing and expressed as cleavable fusion proteins (CDRH3-ScFc or Fab-CDRH3) (step 3). After screening (step 4), TEV protease can be used to excise the knob domain peptides (step 5), which are purified using RP-HPLC (step 6). CDRH3, heavy-chain complementarity-determining region 3; FACS, fluorescence-activated cell sorting; RP-HPLC, reversed-phase high-performance liquid chromatography; RT-PCR, reverse transcription polymerase chain reaction; ScFc, single-chain Fc; TEV, Tobacco etch virus.

In this paper, we present a method for the preparation of an isolated knob domain peptide from bovine antibodies. This comprises immunisation of cattle, fluorescence-activated cell sorting (FACS) of antigen-specific B cells, targeted sequencing of CDRH3 cDNA, reformatting knob and stalk domains for screening, and finally, excision and purification of the isolated knob domains (Fig 2). Most peptides are currently derived by phage display [22] or combinatorial chemistry [23] methods, but the approach described herein is unique in exploiting the bovine immune system to enable affinity maturation and derive peptides with complex networks of disulphide bonds. Even if mammalian display may be used, the structural complexity of disulphide-rich peptides makes their display nontrivial, with the first reported mammalian display of cysteine-rich peptide fragments estimating that just 17% of the library was correctly folded on the surface [24].

For knob domain peptides, the abundance of disulphide bonds may confer improved drug-like properties and render them especially suited for therapeutic applications. The method here is therefore highly applicable to the discovery of peptide therapeutics.

## Results

### Immunisation with C5 yielded an immune serum titre and antigen-specific B cells

**Fig 3:**
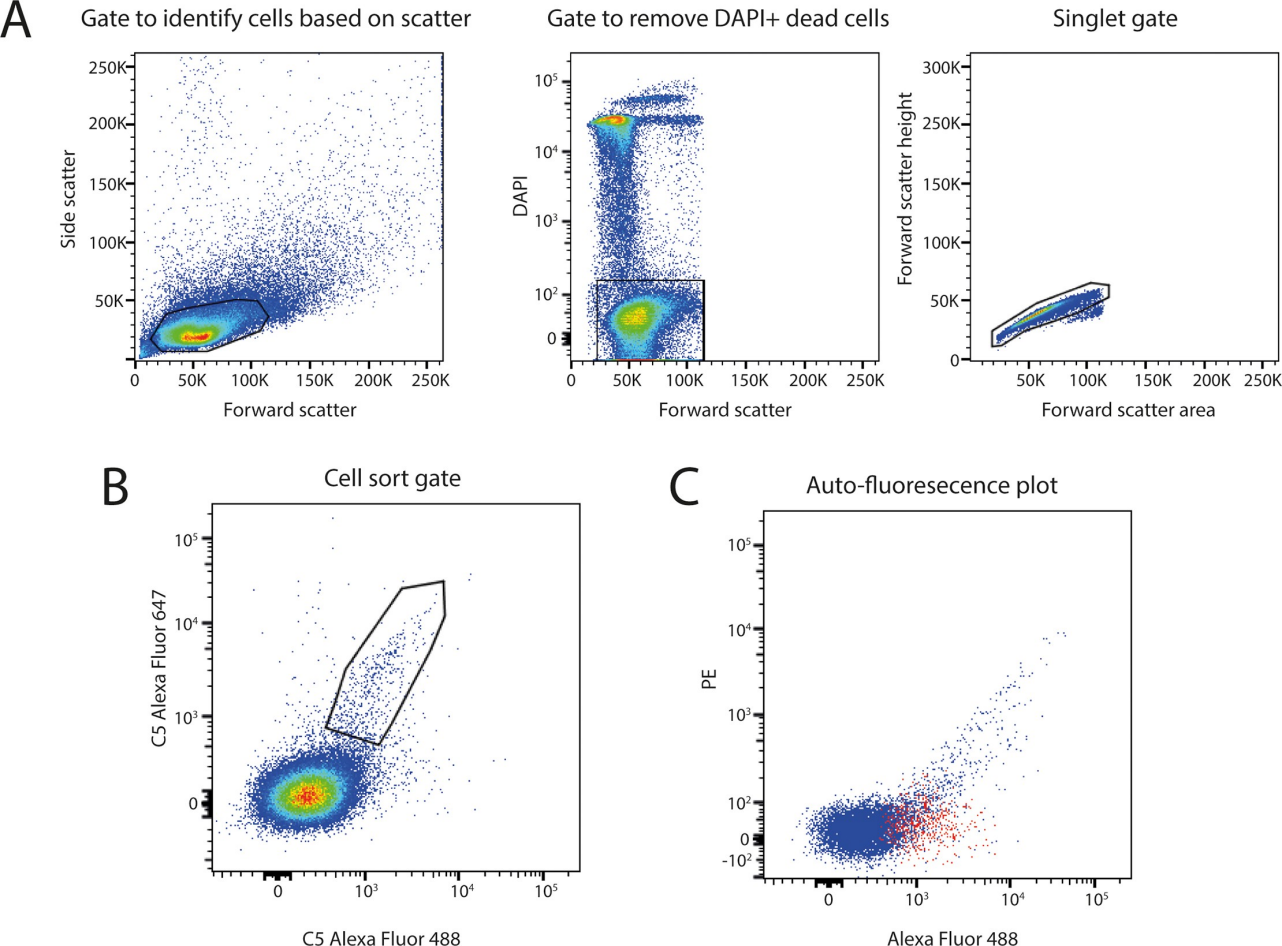
Generation of C5 enriched B cell pool. To identify antigen-specific B cells from immunised animals, FACS of live (DAPI negative) singlet cells was performed (panel A). Two populations of fluorescently labelled C5, C5-AlexaFluor 647 and C5-AlexaFluor 488, were used to identify immune cells that were double positive for C5 (panel B), indicating the presence of surface immunoglobulins against C5. C5 double-positive cells, shown in red, were checked for autofluorescence which may arise in dying or senescent cells (panel C). FACS, fluorescence-activated cell sorting; PE, Phycoerythrin.

We immunised 2 adult Holstein Friesian cattle with human C5 and obtained an immune serum titre of > 1/10,000 (S1 Fig and S1 Data). We isolated immune material from a draining lymph node, taken proximal to the site of immunisation, and performed cell sorting of antigen-specific B cells. Flow cytometry was used to identify cells that were double-positive for 2 fluorescently labelled populations of C5 (Fig 3), and a polyclonal mixture of antigen-enriched immune cells was collected. Similar to camelid VHH discovery from heavy-chain–only antibodies, there was no requirement to pair heavy and light chains, since only the extended CDRH3 knob domains were of interest in this study. Consequently, sequencing of polyclonal libraries of antigen-enriched CDRH3 can be used as a rapid approach to discover knob domains.

### Deep sequencing of target enriched CDRH3 library revealed ultralong CDRH3 clonotypes

The antigen-enriched pool of immune cells was lysed, and reverse transcription polymerase chain reaction (RT-PCR) was performed directly on the lysate. A primary PCR with primers flanking CDRH3, annealing to the conserved framework 3 and framework 4 regions of the V_H_, was used to amplify all CDRH3 sequences, irrespective of their length or amino acid sequence. At this stage, Sanger sequencing of 96 colonies did not reveal any ultralong CDRH3 sequences, potentially as a result of shorter sequences being more readily incorporated into the plasmid vectors. A second round of PCR was used to barcode the CDRH3 sequences for ion torrent sequencing.

Deep sequencing of the CDRH3 library revealed ultralong CDRH3 sequences at 4.3% of the total sequences. Ultralong CDRH3 were easily identified by their length (>90 bp) and by a characteristic duplication of a portion of the immunoglobulin heavy chain variable region 1–7 (IGHV1-7) gene segment that has been reported as a universal feature of ultralong CDRH3 [14]. After filtering, 3,559 unique CDRH3 sequences were obtained from 2 samples taken from a single draining lymph node. Of these, 154 were ultralong. Sequence analysis permitted clustering of the knob domains into clonotypes, based on the homology of their amino acid sequence (S1 Table). A total of 20 clonotypes, displaying sequence identity greater than 75%, were observed. Approximately half of the knob sequences could be classified into clonotypes with the remainder being singletons. A range of cysteine arrangements were present with an even number of cysteines preferred, consistent with the formation of disulphide bonds, and observations in earlier studies [6, 7, 14, 25, 26].

### Ultralong CDRH3 and knob domains were expressed as functional fusion proteins

We attempted to directly express knob domain peptides in isolation using Expi293F cells. From our library of CDRH3 sequences, a small panel of knob domains were synthesised into a mammalian expression vector containing an in-frame 10× histidine (His) tag. Based on the available structures, we designated the first residue of the D_H_2 gene segment, immediately preceding the first germline Cys, as the N-terminal of the knob domain. The C-terminal was the final aliphatic residue preceding the alternating aromatic-aliphatic motif of the descending stalk. In Expi293F cells, knob domains expressed poorly with a C-terminal 10× His tag, so fusion constructs were designed to facilitate expression and purification.

We took the entire ultralong CDRH3 (defined as residues H93–H102 Kabat), comprising the knob and stalk, and expressed with a Tobacco etch virus (TEV)-cleavable, poly-His–tagged, single-chain Fc (ScFc) fused at the C-terminus. A ScFc, where the heavy chains of the Fc homodimer are fused by the incorporation of a linker sequence and expressed as a single polypeptide chain (S1 Text and S2 Fig), was chosen in preference to a conventional Fc to create a monovalent construct [27]. A representative set of 52 ultralong CDRH3, comprising a range of sequences with diverse numbers and arrangements of cysteines, were expressed as CDRH3-ScFc fusions as 2-mL transient transfections of Expi293F cells (S2 Table and S1 Text). Cell supernatants were screened in a C5 binding ELISA, and 14 binders were identified (S3 Table), translating to a 27% hit rate. These binders showed that it was possible to generate ultralong CDRH3 that were viable even in the absence of the supporting infrastructure of the parent antibody.

To assess the importance of the cognate β-ribbon stalk, knob domains were separated from the stalk and inserted into the CDRH3 of a Fab, flanked by TEV protease sites to allow the knob domain peptide to be excised. The human Fab PGT121 was selected as the acceptor framework [28]. The CDRH3 of PGT121, which binds complex-type N-glycans within the gp120 envelope of HIV and has no intrinsic affinity for C5, contains a slightly extended anti-parallel β-ribbon stalk that was an ideal platform on which to present the knob domain peptide.

**Fig 4:**
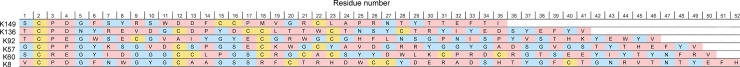
The D_H_ gene segment residues for the knob domain peptides exemplified in this study show the diversity of peptides resulting from bovine immunisation with C5. Residue numbering has been arbitrarily assigned from the beginning of the D_H_ gene segment. To highlight the mechanism for cysteine enrichment within the IGHD8-2 gene segment, cysteine residues are coloured yellow, while residues which may be single DNA base pair change from becoming a cysteine are coloured blue. Of note, the knob domain peptides are different lengths with different numbers and arrangements of cysteines.

**Fig 5:**
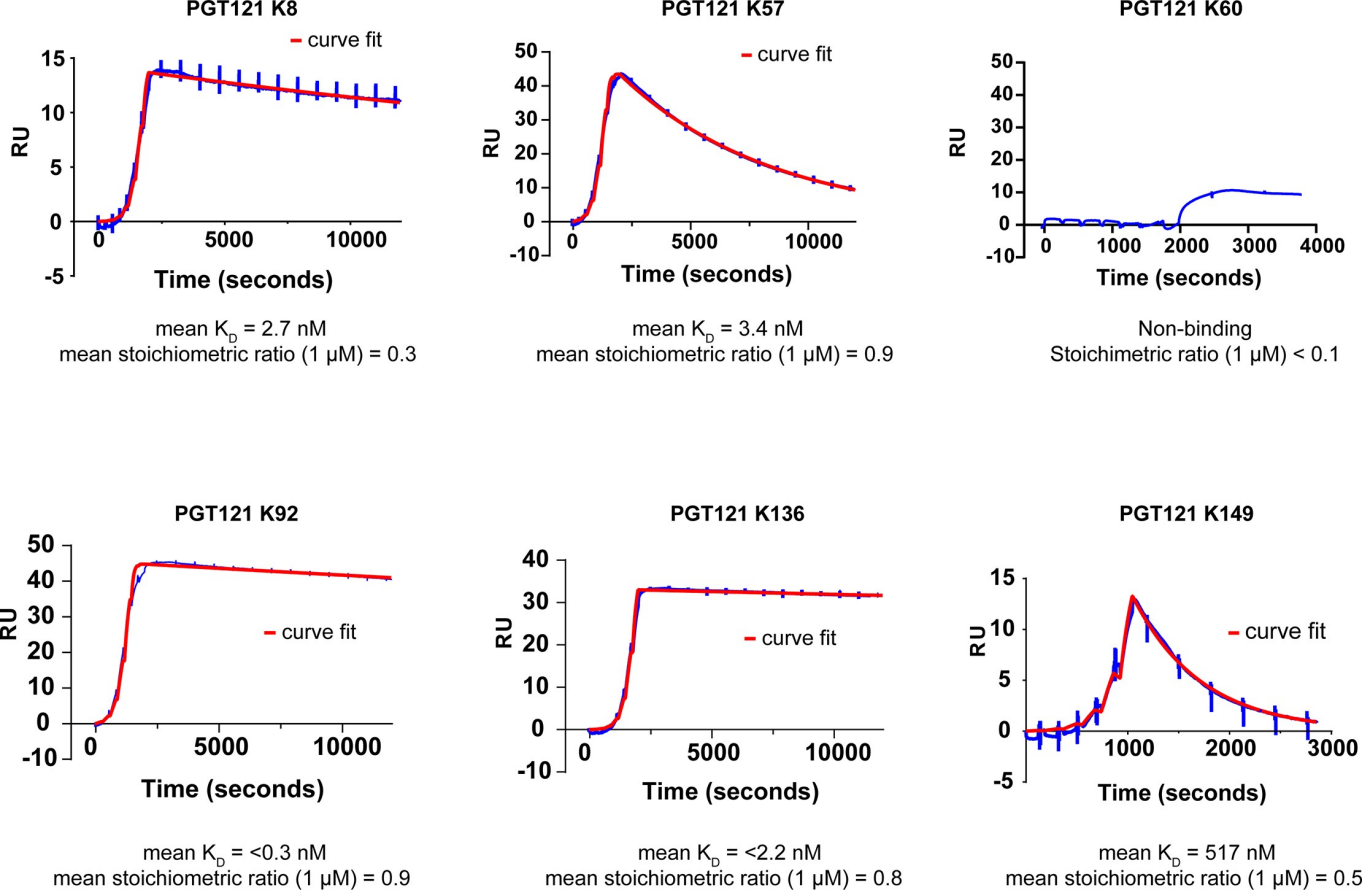
SPR single-cycle kinetics of PGT121 Fab–knob domain fusion proteins binding to C5. As fusion proteins, the knob domains confer high-affinity C5 binding to the PGT121 Fab. A representative sensorgram from Biacore single-cycle kinetics is displayed for each PGT121 Fab-knob domain fusion protein (curve in red), with accompanying curve fits for a 1:1 binding model (in blue). The mean equilibrium dissociation constants (K_D_), determined from 3 experiments, show affinities in the <0.3 nM to 517 nM range for the fusion proteins, except for PGT121-K60, which did not bind C5. RU, response units; SPR, surface plasmon resonance.

From our ELISA hits, 6 diverse isolated knob domains, with different numbers and arrangements of cysteines, were reformatted as cleavable PGT121-knob fusion proteins (Fig 4 and S2 Text). Binding to C5 was measured using surface plasmon resonance (SPR) single-cycle kinetics. C5 has been shown to be activated by extremes of pH [29], and so single-cycle kinetics—in which sequential injections of increasing concentrations of analyte are performed in the absence of harsh regeneration steps—was employed to maintain the integrity of the C5 protein on the sensor chip surface. By SPR, five PGT121-knobs bound C5 with affinities in the picomolar to nanomolar range (Fig 5, S4 Table and S1 Data), with only the K60 knob domain found to be nonfunctional as a consequence of reformatting. Of note, the PGT121-K92 and PGT121-K136 fusion proteins were exceptionally tight binders, displaying off rates that exceeded the 1.0 × 10^−5^ s^−1^ limit for k_off_ determination by Biacore [30].

The PGT121-knob fusion proteins were counter-screened for binding to human complement component C3 (S3 Fig and S1 Data). C3 is the closest human homologue to C5, sharing a conserved fold [31] and a sequence homology of 26.5%. In these experiments, no cross reactivity was detected.

### Isolated knob domains demonstrated high-affinity binding to C5

To obtain knob domain peptides, TEV protease was used to excise the knob domain from a Fab scaffold (Fig 4 and S5 Table). We postulated that the absence of secondary structure and the presence of a stabilising network of disulphide bonds may permit purification by solvent chromatography methods, such as reversed-phase high-performance liquid chromatography (RP-HPLC). RP-HPLC affords greater resolution of minor species compared to size exclusion chromatography (SEC) but is infrequently used for preparative purification of monoclonal antibodies, where milder aqueous-based chromatography techniques are typically favoured. After cleavage, the knob domain peptides were purified by RP-HPLC and freeze-dried. When analysed by liquid chromatography mass spectrometry (LC/MS), masses consistent with the predicted isotope patterns—based on peptide amino acid sequences and formation of disulphide bonds—were present (S6 Table, S4 Fig and S5 Fig).

**Fig 6:**
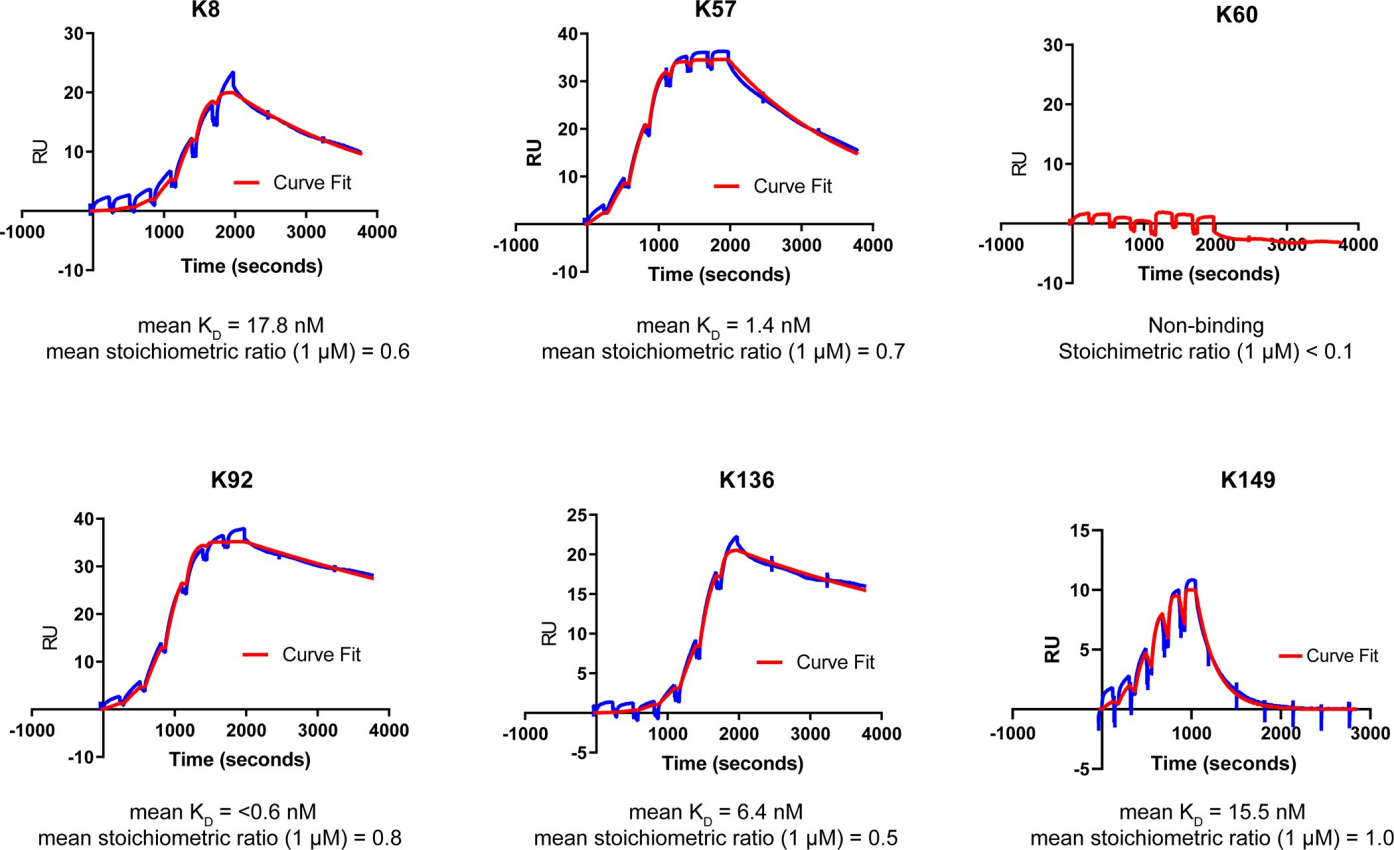
SPR single-cycle kinetics of isolated knob domain binding to C5. The isolated knob domains peptides retain high-affinity binding to C5 when proteolytically cleaved from a Fab scaffold. A representative sensorgram from Biacore single-cycle kinetics is displayed for each knob domain (curve in red), with accompanying curve fits for a 1:1 binding model (in blue). The mean binding affinities equilibrium dissociation constants (K_D_), determined from 3 experiments, show affinities in the <604 pM to 36.5 nM range for all the knob domain peptides, except for K60, which remained inactive after cleavage from the PGT121 Fab. RU, response units; SPR, surface plasmon resonance.

We report individual rate constants (k_on_ and k_off_) and equilibrium dissociation constants (K_D_) for knob domain peptides binding to C5. The peptides display high-affinity binding, with K_D_ ranging from below 1 nM to 40 nM, when measured by SPR single-cycle kinetics (Fig 6, S7 Table and S1 Data). Similar to our experiments with the PGT121 Fab, the only knob domain peptide found not to bind was K60. The knob domain peptides were counter-screened for cross reactivity to human complement component C3 (S6 Fig and S1 Data), and to ovalbumin (S7 Fig and S1 Data), with no binding to either protein detected.

**Fig 7:**
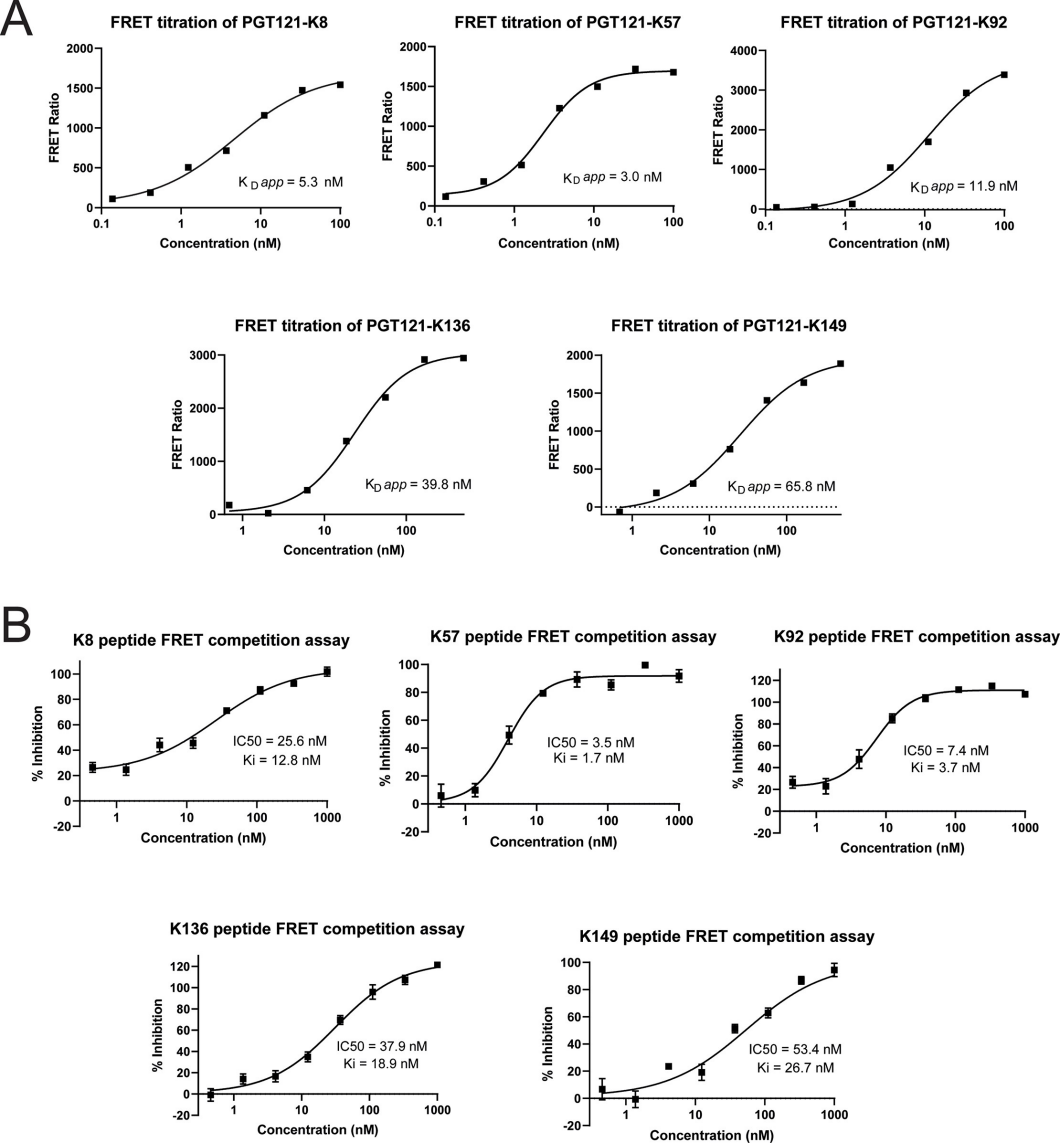
FRET assays. C5 protein was labelled with a Terbium chelate and PGT121 knob domain fusion protein with AlexFluor 647. Panel A shows titrations of labelled knob domain fusion proteins to derive an K_D_ *app* (geomean from 3 experiments). These curves have had background subtracted using quenching concentrations of unlabelled C5. Panel B shows IC_50_ curves obtained by titrating unlabelled knob domain peptide against its respective PGT121 knob domain fusion protein, with the assay performed at the K_D_ *app* concentration. Data are available in S1 Data. FRET, Förster resonance energy transfer; IC_50_, half maximal inhibitory concentration; K_D_ *app*, apparent K_D_.

To provide further evidence for high-affinity binding, we developed steady state Förster resonance energy transfer (FRET) assays. This was achieved by labelling C5 with a terbium chelate donor fluorophore and each of the active PGT121 knob fusion proteins with an AlexaFluor 647 acceptor fluorophore. Titrations of the PGT121 knob fusion proteins—in the presence and absence of a saturating concentration of unlabelled C5 protein—were used to derive apparent K_D_ (K_D_ *app*) for the interaction with C5, after an incubation of 24 hours. Despite modification of both proteins by labelling, the PGT121 fusion proteins bound C5 with high affinity (Fig 7, S8 Table and S1 Data), in a manner consistent with our SPR experiments. Although the PGT121-K92 and PGT121-K136 fusion proteins—which displayed particularly tight binding in the Biacore—displayed lower affinities in the steady-state method.

Next, we tested titrations of knob domain peptides in a competition assay format, using displacement of the parent PGT121 knob domain fusion protein as a readout for binding. For each peptide, the concentrations of PGT121 fusion protein were fixed at the K_D_ *app* and incubated with a titration of peptide for 24 hours. We measured half maximal inhibitory concentration (IC_50_) values and derived inhibition constants (Ki) for each of the knob domain peptides (Fig 7, S9 Table and S1 Data). These data provide further evidence for high-affinity—sub-50 nM—interactions and consolidate our observations by SPR. We note that in all our FRET experiments, the Hill slopes were within the expected range (0.5–2); this is indicative of reversible binding as defined by the law of mass action, while nM displacement of the PGT121 knob domain fusion proteins suggests that the interactions are indeed site specific.

The biophysical and biochemical experiments described here provide the first evidence that isolated knob domains of bovine ultralong CDRH3 can function autonomously, outside of the supporting infrastructure of the V_H_ domain.

## Discussion

Here, we present a method for obtaining isolated knob domains from bovine antibodies, using standard FACS, PCR, and mammalian transient expression. In the present study, we found that knob domains alone were poorly expressed by an Expi293F cell line, but we have shown that they can be readily expressed, either with their accompanying stalk as ultralong CDRH3-ScFc or as just knob domains with cleavable linkers when inserted into a CDRH3 of a conventional Fab fragment. Recovery of isolated knob domain peptides from the expressed Fab fragments was efficient, and readily enabled purification and characterisation on milligram amounts from only a few hundred millilitres of transient cultures.

In the test system we used, 27% of the transposed knob domains from preliminary screening were able to bind to the C5 target protein with high affinity, showing for the first time that they are capable of interacting with antigen autonomously, with neighbouring CDRs not prerequisite for binding. Additionally, we have shown that the cognate stalk is not obligate to orientate or position the knob domain for every bovine antibody. As such, knob domain peptides in isolation constitute a practicable disulphide-rich peptide, being around a third of the molecular weight of a camelid VHH and approximately twice the size of a conventional therapeutic macrocycle.

After immunisation with C5, deep sequencing analysis allowed us to observe 154 distinct ultralong CDRH3 sequences out of a total of 3,559 total unique bovine CDRH3 sequences. In sequencing of naïve bovine antibody repertoires, ultralong CDRH3 are reported to occur at a frequency of approximately 10%, although ranges of 1%–20% have been reported [6, 10, 14, 32–35]. In our antigen-specific CDRH3 pool, the ultralong CDRH3 were observed at a frequency of 4.3%. For the discovery of knob domains, conventional PCR favours amplification of shorter amplicons due to their higher insertion rate [36], which could deselect for ultralong CDRH3. As an alternative, suppression PCR, which favours the amplification of longer amplicons [37], could be explored to try and bias the reaction toward amplification of longer CDRH3 sequences.

From our initial screening with CDRH3-ScFc constructs, we observed a 27% hit rate for C5 binding. While entirely practicable for knob domain discovery, the attrition at this stage may indicate that not all ultralong CDRH3 can function entirely independently of their parent antibody. Previous studies have suggested that the β-ribbon stalk of an ultralong CDRH3 is obligate to orientate the knob domain peptide [6–8, 38]. Inspection of bovine Fab structures in the Protein Data Bank (PDB) frequently reveals stabilising interactions between the knob domain peptide and the β-ribbon stalk, and numerous interactions between the β-ribbon stalk and neighbouring CDRs and V_H_ framework residues [6–8]. In one recent structure (BOV-7, PDB accession code: 6E9U [7]), a disulphide bond was observed between cysteine residues of the stalk and knob domain [7]. Therefore, for certain ultralong CDRH3, removal of the stalk may in turn remove critical interactions required to stabilise the knob domain. Despite this, we have shown that our method can be used to identify isolated knob domains that are capable of binding antigen autonomously, in the absence of the ultralong CDR-H3 stalk region. For the K60 knob domain, which was the only knob domain peptide to become nonfunctional when removed from the β-ribbon stalk, the loss of stabilising interactions of the knob domain peptide and the β-ribbon stalk may have been decisive, markedly increasing the conformational entropy barrier to binding.

For the remaining knob domains, all 5 bound C5 when inserted in the CDRH3 of PGT121, and all were viable binders when proteolytically excised from a Fab scaffold. In the case of the tight binder, K136, a modest decrease in affinity was observed in the isolated knob domain peptide, mediated largely by an increase in the value of k_off_. Conversely, binding of the K8- and K149-PGT121 constructs was slightly improved in the isolated peptides.

We note that although the K_D_ values between the PGT121 knob domain fusion proteins and the isolated knob domain are similar, there are differences in the rate constants. Typically, both the k_on_ and k_off_ are increased in the isolated knob domain compared to the PGT121 fusion protein. In the PGT121 fusion proteins, the N- and C- terminals of the knob domain are constrained by the polypeptide chain and held in proximity, in a manner similar to the native bovine antibody; this might reduce the conformational freedom of the domain and confer stability to the complex. For K_on_, the smaller knob domains might have an advantage in avoiding the steric hinderance which is inherent with a larger scaffold, allowing faster diffusion to buried or transient epitopes.

Our FRET competition experiments provide further evidence that knob domain peptides bind C5 with affinities in the low nanomolar range. The Ki values from the FRET assay are in close agreement with the K_D_ values measured from our kinetic experiments, particularly for the K8, K57, and K149 knob domains, which are within 2-fold. For the K92 and K136 knob domains, which displayed the slowest K_off_ in SPR experiments, the FRET Ki values were approximately 3-fold less than the K_D_ values determined by SPR, but still indicate a high-affinity interaction with C5.

Our data suggest that, for the ultralong CDRH3 population that function autonomously, retaining activity when extracted from parent antibody, the degree of attrition when reformatting from ultralong CDRH3 to knob domain peptide is low. Through our biophysical and biochemical analysis, we have demonstrated that knob domain peptides bind their targets with high affinity akin to a conventional monoclonal antibody, but at a markedly reduced molecular weight, affording an improved ligand efficacy.

Unlike conventional antibody fragments, our study suggests that knob domain peptides can be readily purified by high-resolution RP-HPLC. This apparent robustness, most likely arising from an absence of denaturable secondary structure and the presence of stabilising disulphide bonds, could be advantageous for pharmaceutical development. For example, RP-HPLC can be performed over a wide pH range, allowing pH-driven amino acid modifications, such as deamidation of asparagine [39] and formation of pyroglutamic acid from N-terminal glutamine [40], to be minimised.

Isolated bovine-derived knob domain peptides are novel peptide molecules, with low molecular weight (4–6 kDa) and with intrinsic rigidity and thermal stability, due to extensive disulphide bonding [41]. Having undergone affinity maturation in vivo, knob domain peptides may be more specific for their target protein than peptides derived by naïve display methods. When considered as potential therapies, the reduced conformational freedom, imposed by the additional structural constraints of the disulphide bonds, may enhance metabolic stability [42] and bioavailability [43] and reduce off-target effects [44]. The method described here offers a tractable way to isolate these structures with attractive yields and purity from immunised bovines.

## Methods

### Ethics statement

Bovine immunisations were subject to University of Reading Animal Welfare Ethical Review Body approval (Personal Project Licence: 70/8108 “Antibody Production”) and were conducted in accordance with the UK Animals (Scientific Procedures) Act, 1986 and ARRIVE guidelines, whereby every effort was made to minimise pain and discomfort experienced by animals.

### Immunisation of Holstein Friesians with Complement C5

Two adult Friesian cows were immunised with Complement C5 (CompTech). For early immunisations, 1.25 mg of C5 was mixed 1:1 (v/v) with Adjuvant Fama (GERBU Biotechnik). Three subcutaneous injections into the shoulder were performed at 1-month intervals. Ten days post immunisation, 10 mL of blood was taken to allow testing of the serum antibody titre. Due to a lower than anticipated serum titre, a further 2 immunisations were performed with revised protocols. For subsequent boosts, 1.25 mg C5 was emulsified 1:1 with Freund’s complete adjuvant (Sigma)—and, for the final shot, Montanide (Seppic)—immediately prior to subcutaneous injection into the shoulder. A serum bleed was taken 10 days later to check the serum antibody titre (S1 Fig).

### Harvesting of immune material

Five-hundred-millilitre samples of whole blood were taken, post immunisation with C5. PBMCs were isolated using Leucosep tubes (Griener Bio-one), as per the manufacturer’s instructions. After immunisation, the animals were euthanized, and a draining lymph node from the neck—adjacent to the site of immunisation—and a portion of spleen were collected. The tissues were homogenised using a gentle MACS tissue dissociator (Miltenyi), passed through a 40 μm cell strainer, and collected in Roswell Park Memorial Institute (RPMI) 10% foetal calf serum (FCS). Cells were frozen in FCS, 10% dimethyl sulfoxide.

### Sorting of antigen-specific immune cells by flow cytometry

A sample of draining lymph node was thawed at 37°C and re-suspended in RPMI 1640 Medium, 10% FCS (v/v), 1 mM ethylenediaminetetraacetic acid (EDTA). The cells were centrifuged for 5 min at 400*g* and the supernatant removed. The cell pellet was disrupted and resuspended in Assay Buffer (AB): phosphate buffered saline (PBS), 1 mM EDTA, 1% bovine serum albumin (BSA) (w/v), 25 mM HEPES. The cells were centrifuged as before and resuspended in 2 mL ice-cold AB containing 2 μg/mL each of C5-AlexaFluor 488 and C5-AlexaFluor 647 and incubated for 30 min on ice. The cells were then centrifuged, the supernatant removed, and the pellet washed in ice-cold AB. An aliquot was taken for counting. The cells were centrifuged again, the supernatant removed, and the cells resuspended to 5 × 10^6^/mL in ice-cold AB before filtering through a 40 μm mesh; 4′,6-diamidino-2-phenylindole (DAPI) was added at a final concentration of 1 μg/mL just before acquisition on a BD Biosciences FACSAria III (San Jose) cell sorter. Cells were identified by forward and side scatters, and DAPI-positive dead cells were removed from the analysis. Single cells were identified by pulse processing of height and area scatter parameters. Cells positive for both C5-AlexaFluor 488 and C5-AlexaFluor 647 were then identified and sorted into 1.5 mL Eppendorf tubes containing 1 mL PBS, 20% FCS, 25 mM HEPES kept at 4°C.

The AlexaFluor 488 was excited by a 488 nm laser and collected through a 530/30 nm band pass (BP) filter, and the AlexaFluor 647 was excited by a 640 nm laser and collected through a 660/20 nm BP filter. DAPI was excited by a 407 nm laser and collected through a 450/40 nm BP filter.

### RT-PCR on B cell lysate

The C5-specific B cells from the FACS were pelleted by centrifugation at 10,000*g* in a 4°C centrifuge. The cells were resuspended and lysed with 120 μL of an ice-cold solution of NP-40 detergent (0.5% v/v) and RNasin (Promega) at 1 U/μL. An RT-PCR mix was prepared using Super Script IV vilo Master Mix (Invitrogen), comprising 32 μL of cell lysate and 8 μL of Master Mix. The reaction mix was incubated at 25°C for 10 minutes, 50°C for a further 10 minutes, and finally, heated to 85°C for 5 minutes.

### Primary PCR

A primary PCR was used to specifically amplify IgG CDR3 cDNA sequences. The forward primer anneals to the conserved framework 3 sequence of the V_H_, and the reverse primer sequence anneals to the conserved framework 4 V_H_ sequence. The PCR product when read from 5′ to 3′ therefore encodes the CDRH3 sequence irrespective of length, amino acid sequence, or composition of V-, D-, J- gene segments. The PCR mix was prepared using a Hot-start KOD master mix kit (Merck Millipore), as per the manufacturer’s instructions. The primers used were 5′-GGACTCGGCCACMTAYTACTG-3′ and 5′-GCTCGAGACGGTGAYCAG-3′, and 2 μL of cDNA template was used per 50 μL PCR. The reaction mix was heated for 2 minutes at 96°C and then subjected to 30 cycles of: 96°C for 30 seconds, 55°C for 30 seconds, and 68°C for 60 seconds. Finally, the mix was heated at 68°C for 5 minutes.

### Gel purification

The PCR product contains a polyclonal mixture of CDR3 sequences, comprising regular and ultralong CDR3, which were visualised on an analytical gel. An excision was taken with spanning between approximately 250 and 500 bp, based on the marker. A QiaQUICK gel extraction kit (Qiagen) was used to extract the DNA from the excised portion of the gel, as per the manufacturer’s instructions.

### Barcoding PCR and gel purification

A secondary PCR was performed to barcode sequences for ion torrent sequencing. The primers were as used before but with the addition of adaptor (italics) and barcoding sequence (denoted with **N**): 5′-*CCATCTCATCCCTGCGTGTCTCCGACTCAG***NNNNNNNN**CGGACTCGGCCACMTAYTACTG-3′ and 5′-*CCTCTCTATGGGCAGTCGGTGAT*GCTCGAGACGGTGAYCAG-3′. The secondary PCR was performed using a KOD Master Mix kit, as described for the primary PCR. The secondary PCR product was gel purified and concentrated. Finally, the samples were purified using Beckman coulter AMP magnetic beads, as per the manufacturer’s instructions.

### Ion Torrent deep sequencing of CDR3 libraries

Two samples were prepared for deep sequencing from a single draining lymph node sample from a single animal. The purified DNA samples were diluted to 20 ng/μL (1 μg total) and stored at −20°C. Deep sequencing of all samples was performed using an Ion Torrent PGM technology commercial service by MACROGEN on a 318 chip as previously described [45, 46].

Briefly, approximately 1.1 Gb of data were obtained per chip, which translates to 6,613,415 raw reads of a 170-bp mean length. The first processing step consisted of conversion of the FASTQ to FASTA followed by demultiplexing of the latter to sort the sequences according to their barcodes and to generate individual FASTA files. Each FASTA file was then translated in all 3 reading frames and concatenated into one file containing all frames. Using Perl scripts, the amino acid motifs of the translated primers (DSATYY and LL[V,I]TVSS), which correspond to the conserved framework 3 and framework 4 regions either side of the CDRH3, were used to identify sequences of interest. The number of meaningful reads, after processing, for each sample was found to be approximately 680,000 and 530,000.

### Screening of knob-TEV-HKH-ScFc supernatants

To screen for C5 binding knob domains, ultralong CDR3 sequences were selected based on clonotype, copy number, and cysteine pattern to make a representative set of 52 sequences. Cloning into a pMH expression vector, containing a human cytomegalovirus (CMV) promoter and an in-frame ScFc expression tag (S1 Text and S2 Fig), was performed by Twist Biosciences. For this, and all subsequent protein expressions, a *Mus musculus* immunoglobulin heavy chain leader sequence was used: MEWSWVFLFFLSVTTGVHS (accession number: A0N1R4_MOUSE).

Plasmid DNA for each construct was amplified using miniprep kits (Qiagen). Individual 2.0 mL Expi293F cell cultures (Thermo Scientific), at 3 × 10^6^ cells/mL, per construct, were set up in 48-well culture blocks using Expifectamine 293 Transfection kits (Invitrogen), as per the manufacturer’s instructions. The cells were cultured for 4 days and centrifuged at 2,500 rpm for 30 minutes.

### C5 binding ELISA

Ninety-six-well ELISA plates (Nunc Maxisorp) were coated with a 2 μg/mL solution of C5, purified from serum as previously described [47], in carbonate-bicarbonate buffer (Sigma). All washing steps comprised 4 wash cycles with PBS, 0.05% Tween 20. Blocking buffer was PBS, 1% BSA (w/v). Cell supernatants were plated as 1:10 and 1:100 dilutions in AB (PBS, 0.05% Tween 20, 0.1% BSA [w/v]). To reveal, a 1/5,000 dilution of a goat anti-human Fc, Horse Radish Peroxidase (Thermo Scientific) secondary antibody was used with ‘One-Step’ 3,3',5,5' Tetramethylbenzidine (Thermo Scientific). The reaction was stopped with the addition of a 2% (w/v) NaF solution and the OD measured at 630 and 390 nm wavelengths using a BMG labtech plate reader.

### Protein expression and purification

To enable expression of the PGT121-knob fusion proteins, plasmid DNA was amplified using QIAGEN Plasmid Plus Giga Kits and quantified by A260. Individual Expi293F cell cultures, at 3 × 10^6^ cells/mL, per construct, were set up using Expifectamine 293 Transfection kits (Invitrogen), as per the manufacturer’s instructions. Both heavy-chain and light-chain plasmid DNA were co-transfected at 1 mg/L. The cells were cultured for 4 days, centrifuged at 4,000 rpm for 1 hour, and filtered through a 0.22 μm filter. Using an Akta pure (GE Healthcare), Hi-Trap Nickel excel columns (GE Healthcare) were equilibrated with 10 column volumes (CV) of PBS. Cell supernatants were loaded at 1.0 mL/min, and the column was washed with 7× CV of PBS, 0.5 M NaCl. The column was then washed with 7× CV of Buffer A (0.5 M NaCl, 0.02 M imidazole, PBS [pH 7.3]). Protein samples were eluted by isocratic elution with 10× CV of Buffer B (0.5 M NaCl, 0.25 M imidazole, PBS [pH 7.3]), as 1.0 mL fractions. The column was washed with 0.1 M NaOH and re-equilibrated into PBS prior to subsequent loading. Post elution, the protein containing fractions were pooled and buffer exchanged into PBS, using PD-10 columns (GE Healthcare).

### Cleavage and purification of knob domain peptides

Fab-knob peptide fusion proteins (10 mg/mL) were incubated with TEV protease, at a ratio of 100:1 (*w/w*), for a minimum of 2 hours at room temperature. Peptides were purified using a Waters UV-directed FractionLynx system with a Waters XBridge Protein BEH C4 OBD Prep Column (300 Å, 5 μm, 19 mm × 100 mm). An aqueous solvent of water, 0.1% trifluoroacetic acid (TFA) and an organic solvent of 100% MeCN was used. The column was run at 20 mL/min at 40°C with a gradient of 5%–50% organic solvent, over 11.0 minutes. The column was cleaned with 3 sharp ramps of 5%–95% organic solvent. Fractions containing knob peptide were pooled and lyophilised using a Labconco Freezone freeze drier. For −80°C storage and subsequent analysis, peptides were resuspended with 20 mM Tris (pH 7.4).

### Biacore single-cycle kinetics

Using a Biacore 8K (GE Healthcare), C5 purified from serum [47] was immobilized on a CM5 chip by amine coupling. For PGT121 fusion proteins, flow cells were activated using a minimal immobilisation protocol: EDC/NHS was mixed at 1:2 ratio (flow rate, 10 μL/min; contact time, 30 s). C5, at 1 μg/mL in pH 4.5 sodium-acetate buffer, was immobilized on flow cell 2 only (flow rate, 10 μL/min; contact time, 420 s). Finally, ethanolamine was applied to both flow cells (flow rate, 10 μL/min; contact time, 420 s). A final immobilization level of approximately 100–160 response units was obtained for PGT121 knob domain fusion protein samples.

For knob domain peptides, flow cells were activated using a standard immobilisation protocol: EDC/NHS was mixed at 1:1 ratio (flow rate, 10 μL/min; contact time, 30 s). C5, at 1 μg/mL in pH 4.5 sodium-acetate buffer, was immobilized on flow cell 2 only (flow rate, 10 μL/min; contact time, 420 s). Finally, ethanolamine was applied to both flow cells (flow rate, 10 μL/min; contact time, 420 s). A final immobilization level of approximately 1,500–2,000 response units was obtained for peptide samples. For the PGT121-K149 fusion protein and K149 knob domain, the same immobilisation protocols were used but with a 750 ng/mL solution of C5, instead of 1 μg/mL. This resulted in immobilisation level of 50–85 RU for the minimal immobilisation protocol and 500–570 RU for the standard protocol.

For counter screen experiments, the same minimal and standard immobilisation protocols were used with 1 μg/mL of either C3 (CompTech) or ovalbumin (Sigma), in place of C5. For C3, the minimal immobilisation protocol achieved 40–55 RU of immobilisation. The standard immobilisation protocol used for the knob domain peptides achieved 2,300–2,650 RU of immobilisation for C3 and 330–350 RU for ovalbumin.

One-micromolar stock solutions of the analytes (PGT121 knob domain fusion proteins and free knob domain peptides) were prepared in HBS-EP buffer. A series of six 3-fold dilutions of each stock solution was prepared in the same buffer. The result was 7 solutions for each analyte, at concentrations ranging from 1 μM down to 1.37 nM. A high flow rate of 40 μL/min was used, with a contact time of 230 s and a dissociation time of 1,800 s for knob domain peptides and 10,000 s for PGT121 knob domain fusion proteins. For the PGT121-K149 fusion protein and K149 knob domain, the flow rate was further increased to 100 μL/min. Binding to the reference surface was subtracted, and the data were fitted to a single-site binding model using Biacore evaluation software. Data were inspected for evidence of mass transport limitation using the flow-rate–independent component of the mass transfer constant (tc).

### LC/MS

Knob domain peptides were diluted to 0.1 mg/mL in PBS. Protein LC/MS was performed on a Waters Xevo G2 QTof with a Waters Acquity UPLC.

For chromatography, a Waters BioResolve RP mAB Polyphenyl column (450 Å, 2.7 μm, 2.1 × 150 mm + VanGuard) was used, with an aqueous mobile phase of water, 0.02% TFA, and 0.08% formic acid (FA) and an organic mobile phase of 95% acetonitrile, 5% water, 0.02% TFA, and 0.08% FA. The column was run at 0.6 mL/min at 80°C with a gradient of 5%–50% organic solvent, over 8.8 minutes. At the end of the injection, a sharp ramp to 95% organic solvent and a single cycle of 5%–95% organic solvent used to clean the column.

For mass spectrometry, the system was configured as follows: ion mode: ESI positive ion mode; acquisition mode: resolution; mass range: 400–5,000 m/z; cone voltage: 30 V; capillary voltage: 3.2 kV; desolvation temperature: 350°C; desolvation gas: 1,000 L/h and source temperature: 150°C. Data were analysed with MassLynx software.

### FRET

C5 purified from serum [47] was labelled with an amine reactive terbium chelate (molecular probes, Life Technologies), as per the manufacturer’s instructions. Briefly, C5 at 1.15 mg/mL was buffer exchanged into a 50 mM Bicine, 100 mM NaCl (pH 8.2) buffer using a Zeba column (Thermo Scientific). Terbium was reconstituted in DMSO at 5 mM and added to 1% final (v/v) to create an approximate 10-fold molar excess to C5. After a 1-hour incubation at room temperature, unbound dye was removed by 2 sequential buffer exchanges into 20 mM Tris, 100 mM NaCl (pH 7.4), again using Zeba columns (Thermo Scientific). The labelling ratio was quantified by UV spectroscopy; the final molar ratio of dye to protein was 4:1. Next, PGT121 knob domain fusion proteins were labelled with an amine reactive AlexaFluor 647 (AF647) dye (Molecular Probes, Life Technologies), using the same protocol as above but with a 30-minute incubation with dye. After removal of unbound dye, UV spectroscopy quantified the final molar ratio of dye to protein as 2:1.

For determination of PGT121 knob domain fusion K_D_ *app*, C5 Tb was plated into a black, low-volume 384-well assay plate (Corning) to give a final assay concentration (FAC) of 1 nM; either HBS-EP buffer (GE Healthcare) or unlabelled C5 (1 μM FAC) was added. Serial dilutions of PGT121 knob domain fusion proteins were prepared in HBS-EP buffer to give a range of 100 nM–0.046 nM or 500 nM–0.22 nM (FAC). The plates were wrapped in foil and incubated for 24 hours, with shaking. The plates were read on an Envision plate reader (Perkin Elmer) at 2- and 24-hour intervals (HTRF laser, Excitation 330 nm and Emission 665/615 nm). For fitting, background was subtracted, and curves were fitted using prism software to a 4-parameter logistic model.

For competition assays, C5-Tb was plated to 1 nM (FAC) and serial dilutions of knob domain peptide prepared in HBS-EP buffer, to give a range of 1000 nM–0.46 nM (FAC). PGT121 knob domain fusion-AF647 proteins were prepared to give the following concentrations, which equate to the K_D_ *app* measured in the previous experiment: PGT121-K8 AF647 5 nM (FAC), PGT121-K57 AF647 3 nM (FAC), PGT121-K92 AF647 12 nM (FAC), PGT121-136 AF647 40 nM (FAC), and PGT121-149 AF647 66 nM (FAC). The plates were wrapped in foil, incubated for 24 hours, with shaking, and read on an Envision plate reader (HTRF laser, Excitation 330 nm and Emission 665/615 nm). Curves were fitted using prism software to a 4-parameter logistic model. IC_50_ values were converted to inhibitory constants (Ki) using the Cheng-Prusoff equation [48].

$$Ki=IC50÷\left(1+\frac{\left[R\right]}{K_{D}}\right)$$

in which [R] is the concentration of PGT121 knob domain AF647 used in the assay, and K_D_ is the apparent equilibrium dissociation constant of the PGT121 knob domain AF647 in the assay.

## Supporting information

S1 Fig Serum antibody titres in a C5 serum ELISA. The terminal bleed achieved a titre in excess of 1/10,000.
(PDF)

S2 Fig Diagram of the ScFc construct. A cartoon schematic showing the domain and linker arrangement of the ScFc tag.
(PDF)

S3 Fig Biacore single-cycle kinetics complement component C3 counter screen of PGT121 knob domain fusion proteins. No non-specific binding to C3 was observed with the PGT121 knob domain fusion proteins.
(PDF)

S4 Fig LC/MS on purified peptides, comparison of predicted charge envelopes versus experimental data (1 of 2). 4^+^ charge envelopes are shown.
(PDF)

S5 Fig LC/MS on purified peptides, comparison of predicted charge envelopes versus experimental data (2 of 2). 4^+^ charge envelopes are shown.
(PDF)

S6 Fig Biacore single-cycle kinetics complement component C3 counter screen of knob domain peptides. No non-specific binding to C3 was observed with the knob domain peptides.
(PDF)

S7 Fig Biacore single-cycle kinetics ovalbumin counter screen of knob domain peptides. No non-specific binding to ovalbumin was observed with the knob domain peptides.
(PDF)

S1 Text Amino acid sequence of the cleavable C-terminal ScFc tag. The ScFc is composed of: CH2-CH3-linker-CH2-CH3. TEV site is shown in bold and poly-His tag in italics.
(DOCX)

S2 Text Amino acid sequences of the PGT-121 Fab knob domain fusion. The heavy chain sequences of the PGT-121-knob domain fusions were as follows: the knob domain sequences are shown in italics, with the TEV protease cleavage sites shown in bold.
(DOCX)

S1 Table Clonotyping of 154 ultralong CDRH3 sequences derived from deep sequencing of an antigen specific (C5^++^) pool of PBMCs. The sequences in this table have been derived from 2 samples prepared from the same draining lymph node from a single cow. The first and last residue of each sequence correspond to H93 to H102 of the Kabat numbering scheme, respectively, irrespective of CDRH3 length. Clonotypes are assigned on the basis of ≥75% sequence homology. CDRH3 selected for transient expression and screening as ScFc fusion proteins are highlighted in bold. Sequences marked with a star were characterised as isolated knob domains.
(DOCX)

S2 Table Panel of 52 ultralong CDRH3 sequences selected for reformatting as ScFc fusion proteins. (DOCX)

S3 Table List of 14 ultralong CDRH3 which bound C5 in a single-point ELISA screen. Sequences chosen for reformatting as PGT121-knob domain fusion proteins are shown in bold.
(DOCX)

S4 Table Biacore single-cycle kinetics data on PGT121 Fab–knob domain fusion proteins. Summary of kinetics from *n* = 3. (for individual occasions see S4B Table, S4C Table and S4D Table).
(DOCX)

S5 Table Knob domain sequences derived from Fab cleavage. (DOCX)

S6 Table LC/MS on purified peptides identifies masses consistant with the predicted isotope patterns, based on peptide amino acid sequences and formation of disulphide bonds. Data are shown for the 4^+^ charge state. DSB, disulphide bonds
(DOCX)

S7 Table Biacore single-cycle kinetics data on isolated knob domain peptides. Summary of kinetics from *n* = 3 occasions (for individual occasions see S7B Table, S7C Table and S7D Table).
(DOCX)

S8 Table FRET assay K_D_ *app* for PGT121 fusion proteins binding to C5-Tb. Summary data table for *n* = 3 experiments. For 2-hour data, refer to S8A Table and for 24-hour data, refer to S8B Table.
(DOCX)

S9 Table Competition FRET assays to derive IC_50_ and Ki values for knob domain peptides. Summary data table for *n* = 3 experiments.
(DOCX)

S1 Data (XLSX)

## Abbreviations

AB: assay buffer
aHUS: atypical haemolytic uremic syndrome
BP: band pass
BSA: bovine serum albumin
CDR, CDR: complementarity-determining region
CDRH3: heavy-chain complementarity-determining region 3
CMV: cytomegalovirus
EDTA: ethylenediaminetetraacetic acid
FA: formic acid
FAC: final assay concentration
FACS: fluorescence-activated cell sorting
FCS: foetal calf serum
FDA: Food and Drug Administration
FRET: Förster resonance energy transfer
His: histidine
IC_50_: half maximal inhibitory concentration
K_D_ app: apparent K_D_
IGHD8-2: immunoglobulin heavy chain diversity region 8–2
IGHV1-7: immunoglobulin heavy chain variable region 1–7
LC/MS: liquid chromatography mass spectrometry
PBS: phosphate buffered saline
PDB: Protein Data Bank
PE: Phycoerythrin
PNH: paroxysmal nocturnal haemoglobinuria
RP-HPLC: reversed-phase high-performance liquid chromatography
RPMI: Roswell Park Memorial Institute
RT-PCR: reverse transcription polymerase chain reaction
ScFc: single-chain Fc
SEC: size exclusion chromatography
SHM: somatic hypermutation
SPR: surface plasmon resonance
tc: transfer constant
TEV: Tobacco etch virus
TFA: trifluoroacetic acid
VHH: variable regions of camelid heavy-chain antibodies
VNAR: variable regions of the immunoglobulin new antigen receptor
