# Supplementary figures and images for: Isolation of antigen-specific, disulphide-rich knob domain peptides from bovine antibodies
Source: PLoS Biol. 2020 Sep 4;18(9):e3000821. doi: 10.1371/journal.pbio.3000821 (PMC7498065; doi:10.1371/journal.pbio.3000821)

## Serum Titer ELISA

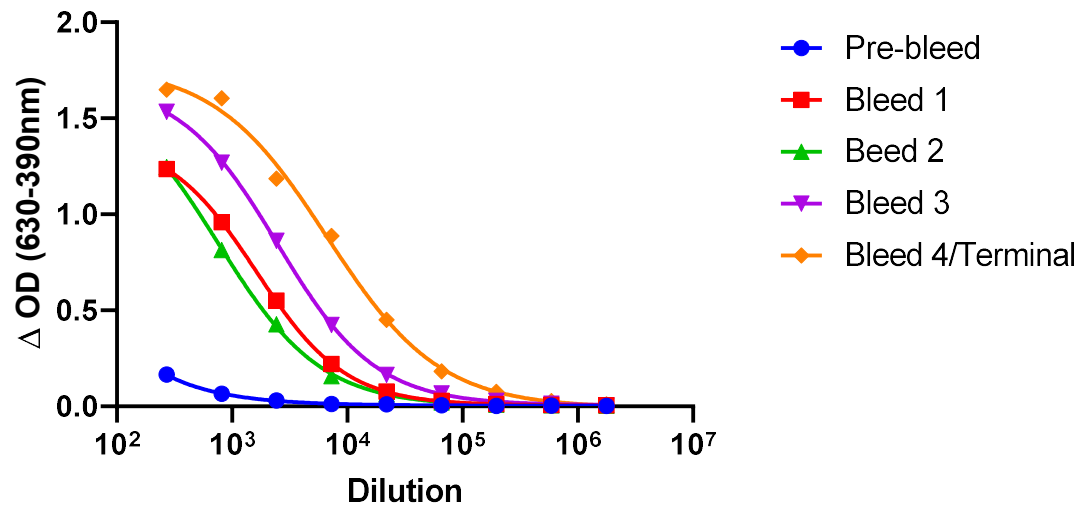

Supplement: S1 Fig — The terminal bleed achieved a titre in excess of 1/10,000. (PDF) [file pbio.3000821.s001.pdf]

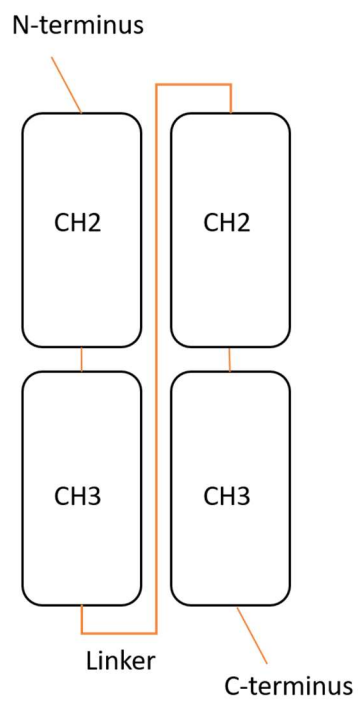

Supplement: S2 Fig — A cartoon schematic showing the domain and linker arrangement of the ScFc tag. (PDF) [file pbio.3000821.s002.pdf]

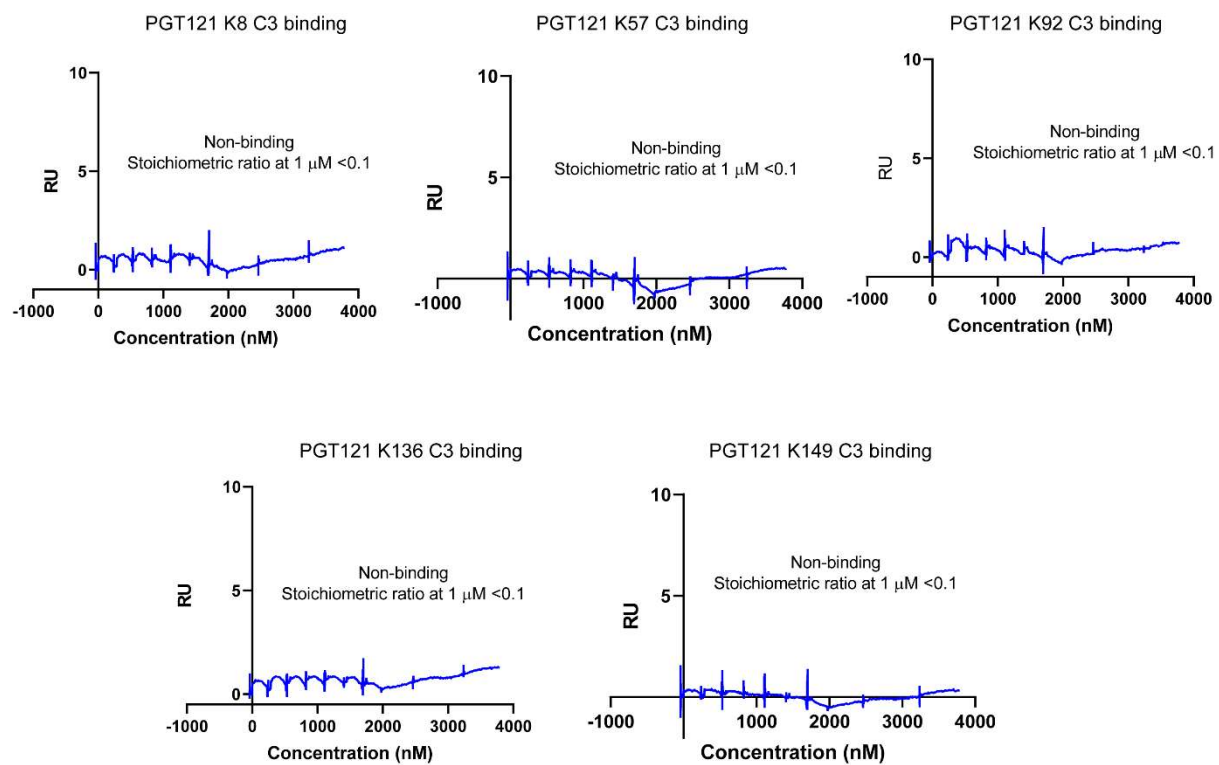

Supplement: S3 Fig — No non-specific binding to C3 was observed with the PGT121 knob domain fusion proteins. (PDF) [file pbio.3000821.s003.pdf]

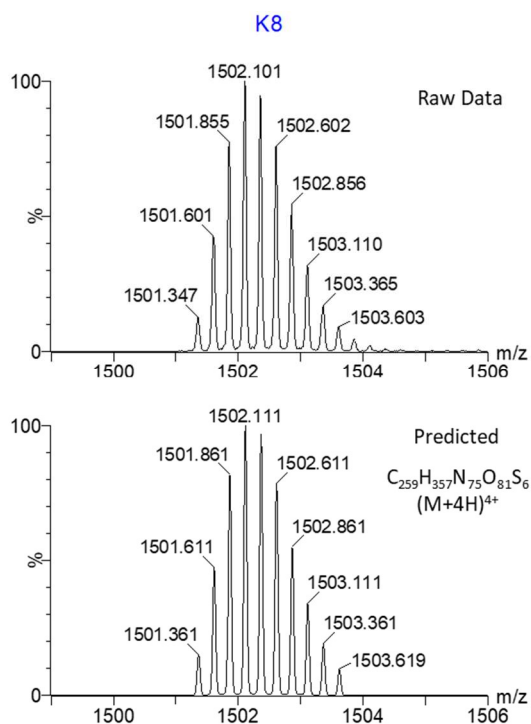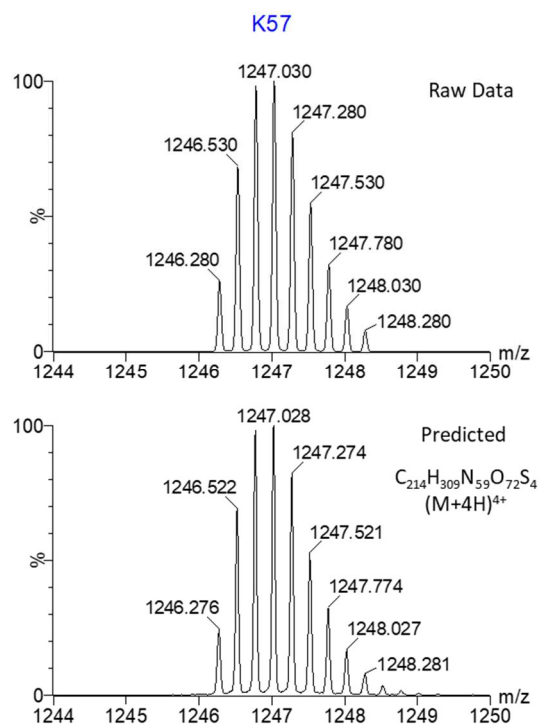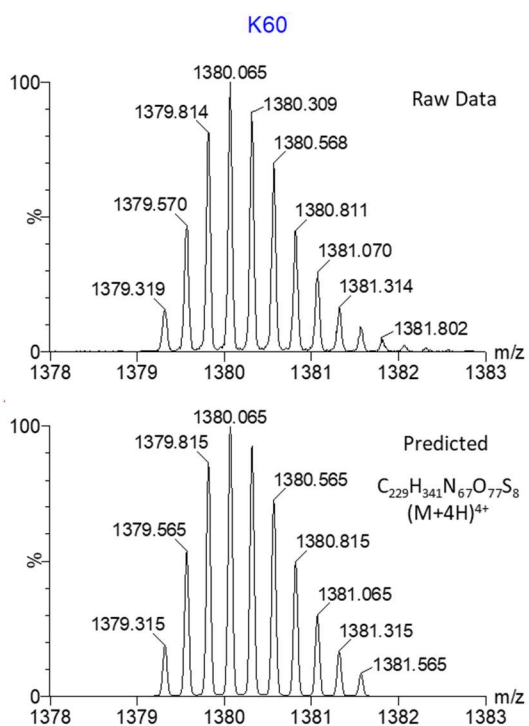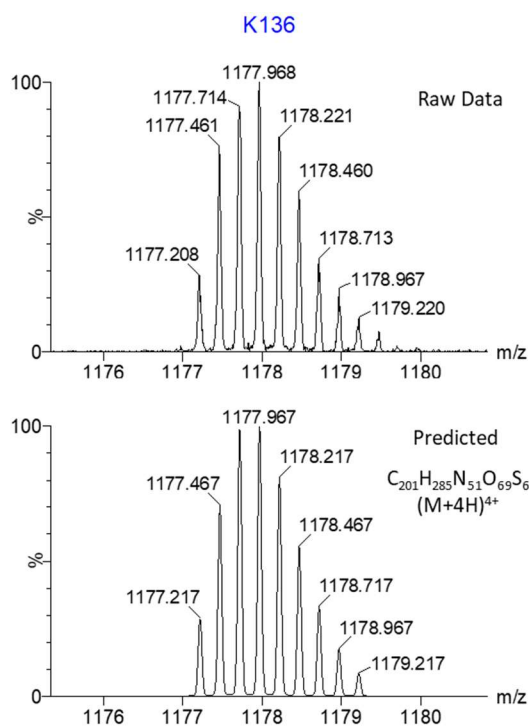

Supplement: S4 Fig — 4+ charge envelopes are shown. (PDF) [file pbio.3000821.s004.pdf]

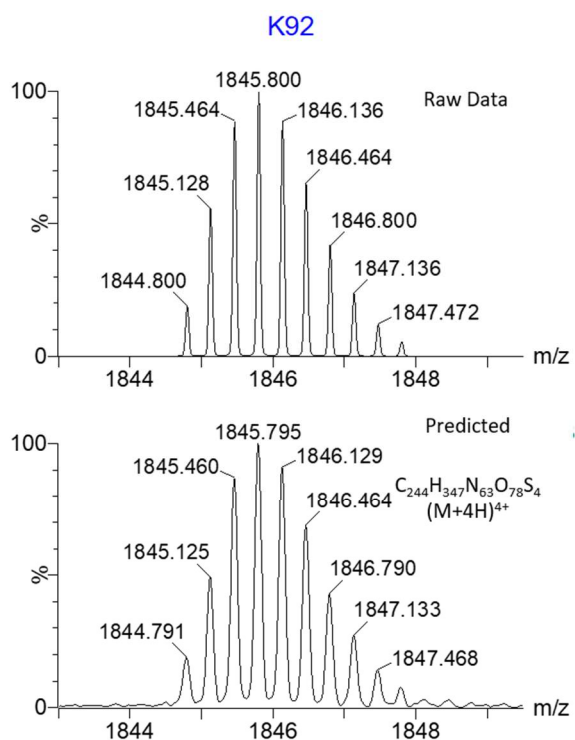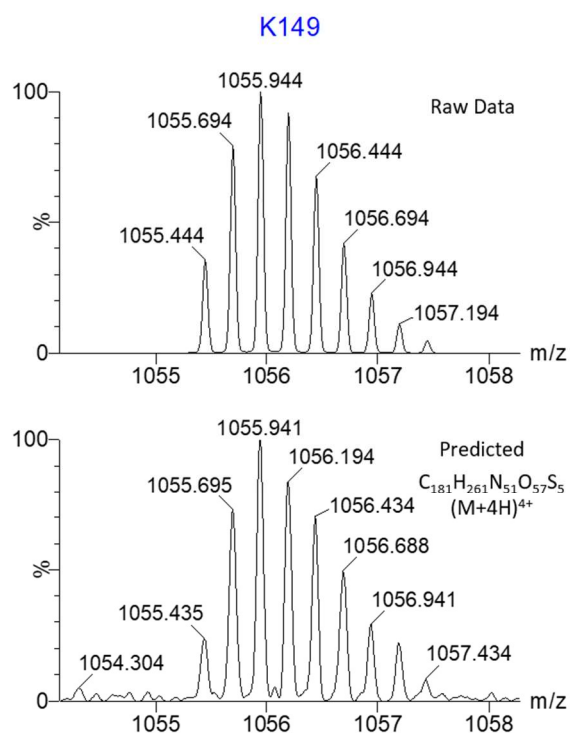

Supplement: S5 Fig — 4+ charge envelopes are shown. (PDF) [file pbio.3000821.s005.pdf]

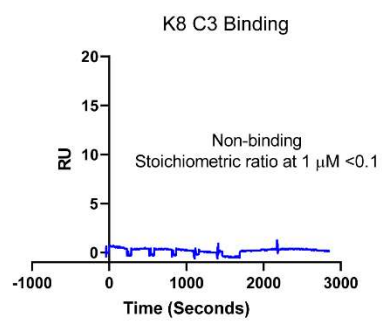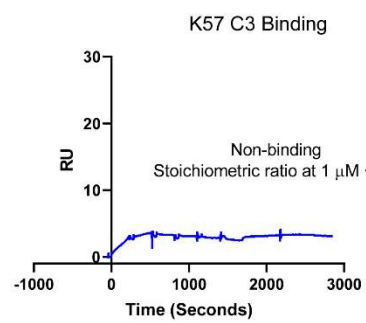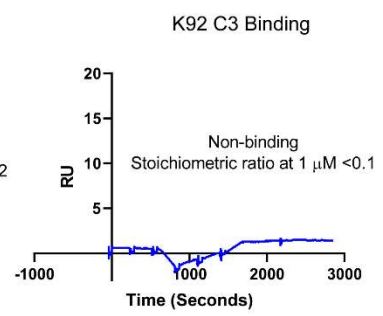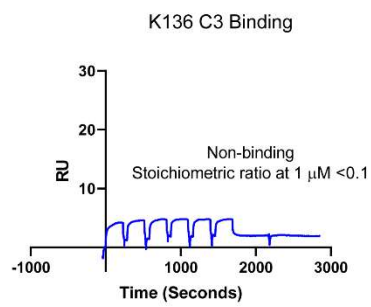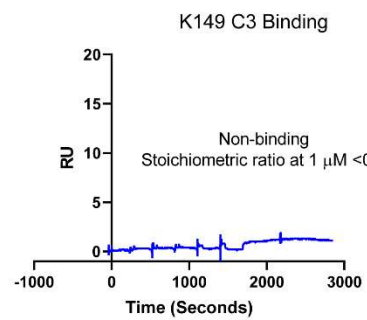

Supplement: S6 Fig — No non-specific binding to C3 was observed with the knob domain peptides. (PDF) [file pbio.3000821.s006.pdf]

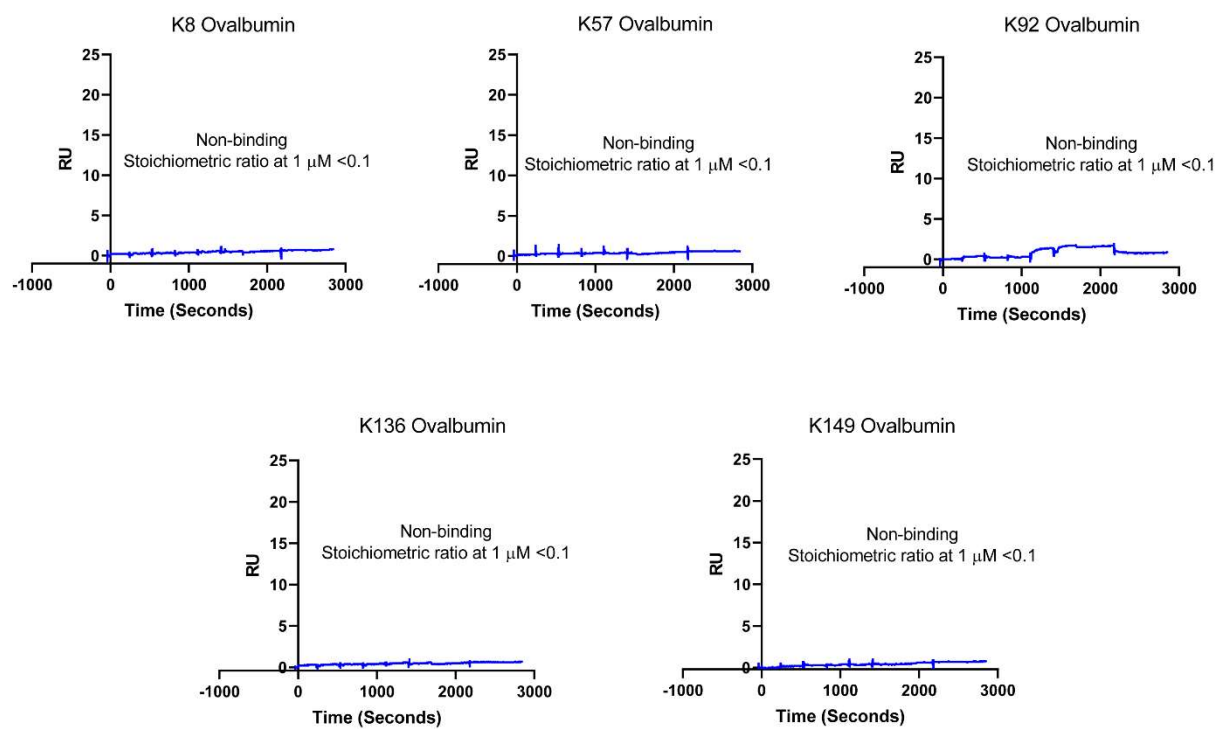

Supplement: S7 Fig — No non-specific binding to ovalbumin was observed with the knob domain peptides. (PDF) [file pbio.3000821.s007.pdf]
