# Supplementary material for: Isolation of antigen-specific, disulphide-rich knob domain peptides from bovine antibodies
Source: PLoS Biol. 2020 Sep 4;18(9):e3000821. doi: 10.1371/journal.pbio.3000821 (PMC7498065; doi:10.1371/journal.pbio.3000821)
Supplement: S2 Text — The heavy chain sequences of the PGT-121-knob domain fusions were as follows: the knob domain sequences are shown in italics, with the TEV protease cleavage sites shown in bold. (DOCX) [file pbio.3000821.s009.docx]

**PGT-121-K149**

QMQLQESGPGLVKPSETLSLTCSVSGASISDSYWSWIRRSPGKGLEWIGYVHKSGDTNYSPSLKSRVNLSLDTSKNQVSLSLVAATAADSGKYYCARTLHGRRIYGS**ENLYFQG***SCPDGFSYRSWDDFCCPMVGRCLAPRN*GS**ENLYFQG**SEWFTYFYMDVWGNGTQVTVSSASTKGPSVFPLAPSSKSTSGGTAALGCLVKDYFPEPVTVSWNSGALTSGVHTFPAVLQSSGLYSLSSVVTVPSSSLGTQTYICNVNHKPSNTKVDKRVEPKSCDKTLEENLYFQGSGGSHHHHHHHHHH

K149 (portion): SCPDGFSYRSWDDFCCPMVGRCLAPRN

**PGT-121-K136**

QMQLQESGPGLVKPSETLSLTCSVSGASISDSYWSWIRRSPGKGLEWIGYVHKSGDTNYSPSLKSRVNLSLDTSKNQVSLSLVAATAADSGKYYCARTLHGRRIYGS**ENLYFQG***TCPDNYREVDGCDPYDCCLTTWCTNSYCTRYI***ENLYFQG**SEWFTYFYMDVWGNGTQVTVSSASTKGPSVFPLAPSSKSTSGGTAALGCLVKDYFPEPVTVSWNSGALTSGVHTFPAVLQSSGLYSLSSVVTVPSSSLGTQTYICNVNHKPSNTKVDKRVEPKSCDKTLEENLYFQGSGGSHHHHHHHHHH

K136: TCPDNYREVDGCDPYDCCLTTWCTNSYCTRYI

**PGT-121-K92**

QMQLQESGPGLVKPSETLSLTCSVSGASISDSYWSWIRRSPGKGLEWIGYVHKSGDTNYSPSLKSRVNLSLDTSKNQVSLSLVAATAADSGKYYCARTLHGRRIYGS**ENLYFQG***VTCPEGWSECGVAIYGYECGRWGCGHFLNSGPNISPYVTT*GS**ENLYFQG**SEWFTYFYMDVWGNGTQVTVSSASTKGPSVFPLAPSSKSTSGGTAALGCLVKDYFPEPVTVSWNSGALTSGVHTFPAVLQSSGLYSLSSVVTVPSSSLGTQTYICNVNHKPSNTKVDKRVEPKSCDKTLEENLYFQGSGGSHHHHHHHHHH

K92: VTCPEGWSECGVAIYGYECGRWGCGHFLNSGPNISPYVTT

**PGT-121-K57**

QMQLQESGPGLVKPSETLSLTCSVSGASISDSYWSWIRRSPGKGLEWIGYVHKSGDTNYSPSLKSRVNLSLDTSKNQVSLSLVAATAADSGKYYCARTLHGRRIYGS**ENLYFQG***SGCPPGYKSGVDCSPGSECKWGCYAVDGRRYGGYGADSGV***ENLYFQG**SEWFTYFYMDVWGNGTQVTVSSASTKGPSVFPLAPSSKSTSGGTAALGCLVKDYFPEPVTVSWNSGALTSGVHTFPAVLQSSGLYSLSSVVTVPSSSLGTQTYICNVNHKPSNTKVDKRVEPKSCDKTLEENLYFQGSGGSHHHHHHHHHH

K57: SGCPPGYKSGVDCSPGSECKWGCYAVDGRRYGGYGADSGV

**PGT-121-K8**

QMQLQESGPGLVKPSETLSLTCSVSGASISDSYWSWIRRSPGKGLEWIGYVHKSGDTNYSPSLKSRVNLSLDTSKNQVSLSLVAATAADSGKYYCARTLHGRRIYGS**ENLYFQG***VCPDGFNWGYGCAAGSSRFCTRHDWCCYDERADSHTYGFCTGNRV***ENLYFQG**SEWFTYFYMDVWGNGTQVTVSSASTKGPSVFPLAPSSKSTSGGTAALGCLVKDYFPEPVTVSWNSGALTSGVHTFPAVLQSSGLYSLSSVVTVPSSSLGTQTYICNVNHKPSNTKVDKRVEPKSCDKTLEENLYFQGSGGSHHHHHHHHHH

K8: VCPDGFNWGYGCAAGSSRFCTRHDWCCYDERADSHTYGFCTGNRV

**PGT-121-K60**

QMQLQESGPGLVKPSETLSLTCSVSGASISDSYWSWIRRSPGKGLEWIGYVHKSGDTNYSPSLKSRVNLSLDTSKNQVSLSLVAATAADSGKYYCARTLHGRRIYGS**ENLYFQG***KSCREGYIDGGGCCLPGSCRGCACSYYDWLKCPRDCRGTSEE***ENLYFQG**SEWFTYFYMDVWGNGTQVTVSSASTKGPSVFPLAPSSKSTSGGTAALGCLVKDYFPEPVTVSWNSGALTSGVHTFPAVLQSSGLYSLSSVVTVPSSSLGTQTYICNVNHKPSNTKVDKRVEPKSCDKTLEENLYFQGSGGSHHHHHHHHHH

K60 KSCREGYIDGGGCCLPGSCRGCACSYYDWLKCPRDCRGTSEE

Heavy chains were paired to the PGT-121 light chain, sequence as follows:

QSVLTQPPDISVAPGETARISCGEKSLGSRAVQWYQHRAGQAPSLIIYNNQDRPSGIPERFSGSPDSPFGTTATLTITSVEAGDEADYYCHIWDSRVPTKWVFGGGTTLTVLGQPKAAPSVTLFPPSSEELQANKATLVCLISDFYPGAVTVAWKADSSPVKAGVETTTPSKQSNNKYAASSYLSLTPEQWKSHRSYSCQVTHEGSTVEKTVAPTECS
