# Supplementary material for: Isolation of antigen-specific, disulphide-rich knob domain peptides from bovine antibodies
Source: PLoS Biol. 2020 Sep 4;18(9):e3000821. doi: 10.1371/journal.pbio.3000821 (PMC7498065; doi:10.1371/journal.pbio.3000821)
Supplement: S1 Table — The sequences in this table have been derived from 2 samples prepared from the same draining lymph node from a single cow. The first and last residue of each sequence correspond to H93 to H102 of the Kabat numbering scheme, respectively, irrespective of CDRH3 length. Clonotypes are assigned on the basis of ≥75% sequence homology. CDRH3 selected for transient expression and screening as ScFc fusion proteins are highlighted in bold. Sequences marked with a star were characterised as isolated knob domains. (DOCX) [file pbio.3000821.s010.docx]

| **CDRH3 Sequence** | **Clonotype** |
| --- | --- |
| **TSVLQSTKPQKSCPDGFSYRSWDDFCCPMVGRCLAPRNTYTTEFTIEA *** | 1 |
| TSVLQSTKPQKSCPDGFSYRSWDDFLLSYGWECLAPRNTYTTEFTIEA |  |
| **VTVHQQTKRTCPRGYEYVSCWWGATCTYGGRCSGSRDDGSLTYEFHVDA** | 2 |
| VTVHQQTKRTCPRGYEYVSCWWGATCTYGGRCSAVGDDGSLTYEFHVDA |  |
| TTVHQEPKKSCPEGYTYVWGCDDDSGGVGYGCAPNGASSCSFTYTYEFHIDA | 3 |
| TTVHQETKKSCPEGYTYVWGCDDDSGGVGYGCAPNGASSCSFTYTYEFHIDA |  |
| TTVHQRTLHNRNCPDGYGYQRHCTVGEDCTERCCDNYGLCTSYTDTYTYEFNVNA | 4 |
| TTVHQRTLKNRNCPAGYGYQRHCTVGEDCTDSCCDRYGLCTTSTETYTYEFNVDA |  |
| **TAVHQRTKRTCPEGLVYNSDQSRCCAADSGVCWEYWRGERVTRGFTYEWYVEA** | 5 |
| TAVHQRTKRTCPEGLIYNSDQSRCCAADSGVCWEYWRGERVTRGFTYEWYVEA |  |
| TTVHQQTHKKRSCPANHSVRDMCSYGPDDCGRSCCTDGIYVRRGSCSSAYEFHVDA | 6 |
| TTVHQQTHKKRSCPENHSVRDMCSYGPDDCGRSCCTDGIYVRRGSCSSAYEFHVDA |  |
| **SIVHQKAHTSVTCPEGWSECGVAIYGYECGRWGCGHFLNSGPNISPYVSTHKYEWYVDA *** | 7 |
| **SIVHQKTQTSEGCPEGWSECGVGTYGYDCGRWGCGHYLNTGPLISGYVTTNKYEWHVEA** |  |
| **STVHQKAHTSVACPEGWSECGVAIYGYDCGRWGCGHFLNSGPNISPYVTTDAYEWYVDA** |  |
| **SIVHQRTQTSKGCPEGWNDCGGNTYGYDCGRWGCGHYLNSGPRISAYQTTYNYEWYVDA** |  |
| TIVHQKTQTREGCPEGWNECGEAIYGYDCGRWGCGHFLNTGPRISGYVTTYSYEWFVDT |  |
| SIVHQKTQTSKGCPEGWNDCGVNIYGYDCGRWGCGHFLNSGPRISAYQTTYNYEWYVDA |  |
| SIVHQRTQTRTGCPEGWNDCGRNTYGYDCGRWGCGHFLNSGPRISDYLTTYNYEWYVDA |  |
| **SIVHQKAHTSVTCPEGWSECGVAIYGYECGRWGCGHFLNSGPNISPYVTTDAYEWYVDA** |  |
| **STVHQKAHTSVACPEGWSECGVAIYGYDCGRWGCGHFLNSGPNISPYVSTHKYEWYVDA** |  |
| STVHQKAHTSVACPEGWSECGVAIYGYDCGRWGCGHFLNSGSKYQSYVTTDAYEWYVDA |  |
| **TTVHQKAHTSVACPEGWSECGVAIYGYDCGRWGCGHFLNSGPNISPYVTTDAYEWYVDA** |  |
| SIVHQKAHTSVACPEGWSECGVAIYGYDCGRWGCGHFLNSGPNISPYVTTDAYEWYVDA |  |
| SIVHQKAHTSVTCPEGWSECGVAIYGYDCGRWGCGHFLNSGPNISPYVTTDAYEWYVDA |  |
| SIVHQKTQTSEGCPEGWSECGVGTYGYDCGRWGCGHYLNTGPLISGYVTTNKYEWHVDA |  |
| STVHQKAHTSVACPEGWSECGVAIYGYECGRWGCGHFLNSGPNISPYVSTHKYEWYVDA |  |
| SIVHQRTQTSKGCPEGWNDCGGNTYGYDCGRWGCGHFLNSGPNISPYVTTDAYEWYVDA |  |
| **TTIQQLTERTCPEGSMLGSECNSHWSCEGCDCAKHCTWGGRCVDCSPYMSTHEWHIET** | 8 |
| TTIQQSTERTCPEGSMLGSECNSHWSCEACDCARHCTWGGRCVDCSPYMSTYEWHIET |  |
| **TSVYQKTDTIRHPCRDDSSYACVCRWTRGCSGTDCSGCTPDSDIDYGCDTIACNYTYQLYVDA** | 9 |
| TTVYQKTDTKKHPCRDDSSYACVCRWTRGCSGTDCSGCTPDSDIDYGCDTIACNYTYQLYVDT |  |
| TTVVPENRHKKHPCRDDSSYACVCRWTRGCSGTDCSGCTPDSDIDYGCDTIACNYTYQLYVDT |  |
| **TTVHQHSNNKKTCPDGTSSHSACILGTGGCCLDQYYRRGICGRVDACYEYSSSVNYEWYVDA** | 10 |
| TTVHQHTNNKKTCPDGSSSHSACKLGTGGCCLDGYYRRGICGRVDACYEYSSSVNYEWYVDA |  |
| **ATVHQRTERSCPDGSSDAESGVCSGCCRGWDCCSFEVDWVGCKGCTAYTYRTVYEHHVDA** | 11 |
| ATVHQRTERSCPDGSSDAESGVCSGCCRGWDCCSFEVDWVGCKGCTAYTYRTIHEHHVDA |  |
| ATVHQRTERSCPDGSSDAESGVCSGCCRGWDCCSFEVDWVGCKGCTAYTYRSIYEHHVDA |  |
| VTVHQRAERTCPDGSSDAESGVCSGCCGGWDCCSFKVDWVGCKECTAYPYNTRYEHHVDA |  |
| **TTVHQQTKTKKNPCRDVASPVCVCRWAEGCSGTDCSECTPDPDRDYGTCEIIACTHTYELHVDA** | 12 |
| TTVHQKTKTKKNPCRDVTSPVCVCRWAEGCSGTDCSDCTPDPDRDYGTCEIIACTHAYELHVDA |  |
| TTVHQKTKTKKNPCRDVTSPVCVCRWAEGCSGTDCSDCTPDPDRDYGTCEIIACTHTYELHVDA |  |
| **TTVIQKTATKQSCPDDYRDGGECCIYGRCSAEDCSVTGWEYYGSTLCRVPYITTHAYQWHVDA** | 13 |
| TTVIQKTATKQSCPDDYRDGGECCIYGRCSAEDCSVTGWEYYGSTLCRVPYITTHSYQWHVDA |  |
| TTVIQKTATKQSCPDDYRDGGECCIYERCSAEDCSVTGWEYYGSTLCRVPYITTLAYQWHVDA |  |
| TTVIQKTATKQSCPDDYRDGGECCIYGRCSAEDCSVTGWEYYGSTLCRVPYITTLCLPVARRA |  |
| **TTVHQETRRNCPDGYSEINACGDRYKASGGLCCGEGAGAWRCWECSDTIIPTTTYEFYVDA** | 14 |
| **TTVHQETRRHCPDGYSDIYGCGHYYSATGGHCCGEGAGAWRCWECSDTIMPSTTYEFYVDA** |  |
| **TTVHQETRRNCPDGYSDIYGCGNRYAATGGHCCGEGAGAWRCWECSDSIWPSSTYEFYVDA** |  |
| STVHQDTRRHCPDGYSDIYACGHYYSATGGHCCGEGAGAWRCWECSDTIMPSTTYEFYVDA |  |
| TTVHQESRRHCPDGYSDIYGCGHYYSSTGGHCCGEGAGAWRCWECSDTISPSTTYDFHVDA |  |
| STVHQDTRRHCPDGYSDIYGCGHYYSATGGHCCGEGAGAWRCWECSDTIMPSTSYEFYVDA |  |
| TTVHQETRRNCPDGYSNIYDCGHYYSSSGGHCCGEGAGAWRCWECSDTISPSTTYEFYVDA |  |
| TTVHQETRRSCPDGYSDIYGCGHYYSSTGGHCCGEGAGAWRCWECSDTISPRTRYEFAVDA |  |
| TTVHQETRRNCPDGYSDIKGCGNAYAATGGHCCGEGAGAWRCWECSDTIAPSSTYEFYVDA |  |
| STVHQETRRSCPDGYSDIYGCGHYYSSTGGHCCGEGAGAWRCWECSDTISPSTRYEFYVDA |  |
| TTVRQETRRNCPFGYSDIKGCGNRYAATGGHCCGEGAGAWRCWECSDTIRPSSTYEFYVDA |  |
| **VIVYQETIKSCREGYIDGGGCCLPGSCRGCACSYYDWLKCPRDCRGTSEEYIYTYNFRVDA *** | 15 |
| GIVYQETIKSCPEGYIDGGGCCLPGSCRGCACTYYNVLKCPRDCRGTSEEYIYRYKFHVDA |  |
| **STVHQLTITTLGCPDGVSVVNTCGWLRCNCGDSIYCSRSADSGMWCGRCGDCTSTHTHQWHVDA** | 16 |
| **STVHQLTITTLGCPDGVSVVPTCGWLRCNCGEDLYCSRSDEQGTWCGRCGDCTSTYTHQWHVDA** |  |
| **STVHQLTITTVGCPNGVTRVATCGWKRCHCGENIYCSRSDDSGTWCGRCGDCTGTYTYQWHVDA** |  |
| STVHQLTITTVGCPNGVPRVTTCGWKRCHCGENIYCSRSDDSGTWCGRCGDCTGTYTYQWHVDA |  |
| GTVHQLTITTLGCPDGVSVVNTCGWNRCNCGDTTFCSRSDDSGTWCGRCGDCSSTHTHQWHVDA |  |
| STVHQLTITTLGCPDGVSVVNTCGWKRCNCGDSIYCSRSADDDGWCGRCGDCTSTHTHQWHVDA |  |
| STVHQLTITTVGCPNGVTRVATCGWKRCHCSENIYCSRSDDSGTWCGRCGDCTNTYTFQWHVDA |  |
| **TTVHQKTIAKCPDGYTYSGDCGICDDCGGRTSRAYDCAGDTSLYMCGRRSPTLLTYQFHVDV** | 17 |
| TTVTPETIAKCPDGYTYSGDCGICDDCGGRTSRAYDCAGDTSLYMCGRRSPTLLTYQFHVDV |  |
| **ATVHQQTKKQTERSCPDGYTYINDCIGASGAVSRYDCWRFRRMNGVCIDGTYSTTADTYTYEFHVDA** | 18 |
| ATVHQQTKKQTERSCPDGYTYIVDCIGATGAVSRYDCWRFRRMNGVCIDGTYSTTADTYTYEFHVDA |  |
| **TTVHQKTRKSCPGGCRDTDGHDYDHWSCAGSDCCCFGTDGGCGRWGIYCSHSYTYTYEYHVET** | 19 |
| TTVHQKTRKSCPGGCRDTDGHDYDHWSCAGSDCCCFGTDGGCGRWGVYCSHSYTYTYEYHVDT |  |
| TTVHQKTRKSCPGGCRDTDGHDYDHWSCAGSDCCCFGTDGGCGRWGIYCSHSYTYTYEYHVDT |  |
| **CTVQQKTHQVCPDGFNWGYGCAAGSSRFCTRHDWCCYDERADSHTYGFCTGNRVTNTYEFHADA *** | 20 |
| TTVQQKTHQDCPDGFNWGYGCAAGSSLHCARHDWCCYDDRVGRDTYGFCTGNRATTTYEFHVDA |  |
| **TTVHQKTDQKRSSCPDGYSDCLVCGADRDGCSSGGCRGCWTNAYYSSRTYYNTDEFHYKPNEFHVDM** | None |
| **TSVYQKTTKRFTCHDPSGGTWERADGATSCPGTHCCSYGRDGIWHGYDRRRTYTEVFTYELDVEE** | None |
| **CTVYQKTETKKSCPDGYRFFQECRGTGTGCPGDDCVCYDGRGGFRWRNGCTTYTYTYRHNLHVET** | None |
| TTVYQETKIMRICPDDERRRWGCSDDSEGCSDSDCHIYDGDGSVGCCDGYLNSREIYKYAFHIDA | None |
| VAVHQKTTERYSCPDGYSSCSSCRANDLDCRGVDCVNDRVCRGDGGFFSSRGYIVTYNYDFRVDA | None |
| AAVHQETKTLRTCPPGLSDSNACPVGTWASRRTGCCSCCDRFCGGYSTCTDYTDTYTYEWHVDT | None |
| TTVHQETKITSPACPDGYFYEYRCLVGGGCGWGCWNAAGGRPNAAGSLDRSPIETVTYEFQVDA | None |
| TTVYQKTTKSTCPDGYIADGGCRKAGSWCSSVDCAGYGEDGDYGGWRTSCCYFVASAYEFHVDT | None |
| GTVHQQTQEKCPDGYTFTANNCVTSSVRCSGRNCCGGDSYGYYIGIGGICHYDYTYTYENYVEA | None |
| **TIVHQETNKEKICRVDYVDSATCTWNCDCCRSRKSDCCAYANSRSCWNTSGTYTYTYEFHVDA** | None |
| **TTVHQRTITRCPDDFGNTCRCSKGTCPCGEDACCGTNQYSFWGDCRDVGRTTFIETYEWNVDD** | None |
| **TTVYQNTRSKERSCPYGTGFDPTWCDSVLPCRRDGCWTTVWGCCEGDVDGGETTPTYEFYVDA** | None |
| **TTVYQKTRSDCPAGYKQVYGCSAGNCGCRGNGCCNSGSCGTWSEWGQYGCCNCHSSYEFHVDA** | None |
| **TTVHQTTRKTQSCPDGYTDIDGCSWRHGCCRYDCCSDRSCSWCVDRDWSSYIVTATYELDIEA** | None |
| TTVHQETKHTRSCPDGYTDRVGCPYLWTSCARGDCWRIDRGATANPAATTYTYTDTYDWHIET | None |
| **ITAHQKTNKIPHCRDGYDYGGGCCVSSGVYGESCRSSGGSDCDQWVGCESVTYTETYEWHVDA** | None |
| TSVLQKTRHTCPDGYEYDTACGHGRCCCVGSSCRRNHTYGDYRRWGLYNSYSPAYTYEFHVDT | None |
| **TAVHQQTERSCPPDTTEHDCCGCGGRGCAWSGCYRKGYGTGCRVCTSIQARDYIYTYKLHIDT** | None |
| TTVHQNTIRSCPDGTDYAYGCRLGAWGCAGVGCCRGGAVGAWGCYGGDTFNTDSYTYEFYVDA | None |
| TAVYQRTEARKSCPDGYNDVEARAHRSECSPNDCLRDGLGVASGCAWYRAYILIETYEFYVEA | None |
| TTVYQKTRKLPSCREGTFYHAVCGGVVRCQVVDCDADGGCCYNAIGQYFGVSYSYKYEWFVEA | None |
| ATVHQKTNKKQSCPDGYSDDDGRPDHWSCMDVDCWRPARGGWGSNCEHTNYIYTYTYEYHVDA | None |
| **TIVHQKTKREERCPAGYSISACRDGIGCGATDCCADGATDYAWGWECKSRIYGDSYEFHVDA** | None |
| TTTVQRTHKTTSCPDGYHFIEPCHSGLCWREGACNGDGICANGLGRCRTVSETSTYEFYVDA | None |
| TTVYQRAQSKSCPDYCSCIFSYCSGADGCSSYGYCGHGGDEGDGFNGGGSRVSYTYEFYVDS | None |
| TTVHQQTRTRCPDDYSYRSRGWIGSDCGGHGCWSDRDARRYDVYGNCNRVGEINTYEWYVDA | None |
| TTVHQRTKKKLVLSVMILMIVVTILILCRVEECCKNGVVNAYGICEYAGGSATYTYEWYVDA | None |
| **TTVHQKTITSCPDGYVYSYDCGICDDCGGRTSRAYDCAGDTSLYMCGRRSPSSAYQFHVDR** | None |
| **TTVHQRTIKSGCPPGYKSGVDCSPGSECKWGCYAVDGRRYGGYGADSGVGSTYTHEFYVDA *** | None |
| **TTVHQRTKKTCPLGYDLNDRCDHFNTCRVEECCKNGVVNAYGICEYAGGSATYTYEWYVDA** | None |
| TTVHQKTQRPICPDDYTALNGWGCGEYRCCPKSGACCCSGGGVHLLQSCSLETKYEFYVSA | None |
| LTVLQVTDRRASCPAGCQDECGSSENCYCFRYGIWCHGRYSSGNSGTYSSNGYSSTWYADA | None |
| STVHHEAHKRCPEDYSDRDHCSCWAGCGDDDCWRVVAGWRCSNYRYIGASYTHTYDFYADT | None |
| **TTVHQKTKKSCPLGYAINDRCDDLKTCGPDECCLNGVVNAYGICEYEGESATHTYEWYVDA** | None |
| TLVYQKTKKSCPEGYEGAPDCGAFDYCRVDDCCCRSGYGSCRRDSCRSGIRTSTYEFYVDT | None |
| TTVYQHTRNRCPDDYRDCGHCCCQYGCHAVGCWRRQGGGFERCGEVDSQSPTYMYEFHVDA | None |
| ATVLQYTHKTCPDGYEFGKNCPDGHGCSGSDCWRCDSRSAWWCTNYSWTDSIHAYELYVDA | None |
| TTVHQKTEKSCKGGTDCGAGCCADGDPCSSGRCRAWSSTLRDYFYYPTSNYTYICDFHIDA | None |
| TIVFQKTTKSCPGVSAEGGVCCSGTACTVPECWWFHQGHYSIPGGCTAATYTHTYESHVDA | None |
| TTVHQKTNQEKHCPDGYDYCRVTEDGYCCSAWTCMHWRCAPGHKEYSVVSTTYTYEWYVDA | None |
| VTTHQKSRKICPDGCIYACSCREEWRCTVFDCVRPRDVPNGRNACVSTCPSTSIYEFRVDA | None |
| **STVYQETKRKCPDGYRVGTDCTPGKGCDYACHSRLGVRWGGDGRDGGRGYIVSYELHIDA** | None |
| TTVYQKTQKPTDGYSCGITCRKRCDCSFVGYCACSESVSGDCTCYPRDSIPYRHEWYVDA | None |
| TTIFQKTRRNCPPSSTSDGDCRGGWTCRGGDCSRWRGYYSSGNNYCCYDYTDTYEFYVDQ | None |
| TTVRQKTAKSCPWGYDNGHGCNCGNDVFACSECLRSGTCSRYGRYEAYSYIVTYEFSVDA | None |
| STVHQKTRQSCPDDYPVKCERGCGRERCGNCGWACNGPVGSPTCSYCRPYIYTYEFYVDA | None |
| **TTVYQKTKETCPDGYIWAERCPGGWTSCRNACWLEGGDSAGAYDEVTSTVHRYEFYVDT** | None |
| TAVYQKTEEKNTCPDGHTWRHGYRCTGWSYGCFRGAGNDCSDFGGDRITTYGYEWYVEN | None |
| TTVHQRTIRTCPDGYGYQDACGRWGGCVGRACCSSGGSGCCDGSCGTMYIDNYDLYVDA | None |
| **GIVVQRTYERRTCPDTFTYKDGCRRGGTLLNSRSGCYNVYCNYHDAEVTYAHRWYVDA** | None |
| **VTVQQQTKLEYSCPNGYSSDAGCLAAWRCGDYDCCRENAFRPCTGSIPTSNYEWHLEA** | None |
| STVHQETIRTCPDGGTYARDCGRECAICGHCGCCQNAYRRNWETCNTYTESINFHIDA | None |
| TAVVQRSLKKPSCPSGYTLWGDCEGDDGGEGGVCRCWRPHSTVATPTYASTFQWHVDA | None |
| TTVYQKTNTERSCPEWVQTSRTCIYRSRCGQYVCWSLGEDDCGVTCTDTTTYEWYVDA | None |
| ITVHQETIRTCPDAWRSSATCRGAYGEAYECCPSGSSMWTSCVGCTTATFSYNLYVEA | None |
| TTVHQKTSAKRTCPDGWRPGSECGWEDRCCGEFCSRCDWHGGWRAYMETQTYEFNVDS | None |
| STVHQQTNKRRQNCPDGYKYNGFCTPDGGCSRVSSWGWDRSCISPTYTYTYEWYVEA | None |
| **TTVYQSTRKTSRNCPDGGSPSVQCLDDTWACRIVDCYDDGTYGTYRFTNTYDWYVDA** | None |
| STVFQHTKTTCSCPDNWETSGDCAGSSGDCSDCTCWRLGYGRTSSIATFNYEWYVEA | None |
| TTVHQKTESHRSCPGDRPVDCGDDYGTLGCCPFHVGCGTWRCIEHIYTYTYQFHVDA | None |
| TTVYQSTRKTSRNCPDGGSPSVQCLDDTWACRIVDCYATVLMVPIVLPTLYDWYVDA | None |
| ATVHQYTHRSCPVGYDGGGNCGRYVDTCWGSDCCRYRRGIDYSCSSYSSSYEFYFEA | None |
| ASVHQETKRSCPDGYRRGLECSAEWRCRYYDCVECSYGLCGHITRYIESYAWHVDT | None |
| TAVHQETKKQPPNCPDGSSLLSSCFDTGGCSLYSCGREGRRRTYTYSYTYEWYVDA | None |
| GTVHQKTNDHTRCPDGYYQGWHMSLRRYVCARDGYNPERYYVEATHTYTYEFHIDA | None |
| TTVHQQTNTKNCPTWCGFAHSCILRYEACSDCDCSGGAGDYAAPGLYHTYEFHVDA | None |
| IAVHQETKRSCPGGYIARCAGTYGCSAVPGCCDFSGDCLWRADSLTLTYELHVDT | None |
| ATVHQKNNRKKKLVRMVVNLVSSVSTPVKFCRISECYEDHPTTIYTYTYEFHVDA | None |
| **TTVHQTTNRKKTCPDNYREVDGCDPYDCCLTTWCTNSYCTRYIYEDSYEFYVTA *** | None |
| **ATVHQKTTEKKTCPDGGEPSVICLDASEVCRISECYEDHPTTIYTYTYEFHVDA** | None |
| TTVHQKTKRSCPAYDSSGCGCVYYSPWNACICDKPGGPCDGVNPITSYEFNVDA | None |
| TAVYQKTSESQRTCPSWCSLYMCGGYLACSACGCAENGRYGNGITYTYEWHVDA | None |
| TTVHQKTTKTCPDGYVYNDPCDCWGRRNYDCCCEGGREFYTFVYSHEFNVHS | None |
| **TSVLQSTKKQKSCPDGLSYRAWDDFCCPNVGRCLPPINTYTYTHAFHIEA** | None |
| **TTLYQNTRKKGGCPEGTTYLGGSSETYRCGLEGRMRTYSYTYSYEWYVDA** | None |
| TSVLQSTKKQKTCPDGLSYRSWDGFCCPKVGRCLPTIDAYINQFHIEA | None |
| TSVLQSTKEQKTCPDGLSYRSWDGFCCPKYGRCLAATSTYTTEFYIEA | None |
| **GAVYQKTNEQSSCPDGWRDTGTHCEDYGSWGYRDYTFTYTYEFHVHN** | None |
| TTVHQTTRPNTDSCPSGYSTTLHCCCGSWKCDWCDPTTYKYELYVNA | None |
