# Supplementary material for: Isolation of antigen-specific, disulphide-rich knob domain peptides from bovine antibodies
Source: PLoS Biol. 2020 Sep 4;18(9):e3000821. doi: 10.1371/journal.pbio.3000821 (PMC7498065; doi:10.1371/journal.pbio.3000821)
Supplement: S2 Table — (DOCX) [file pbio.3000821.s011.docx]

| **Sequence** |
| --- |
| TSVLQSTKPQKSCPDGFSYRSWDDFCCPMVGRCLAPRNTYTTEFTIEA |
| VTVHQQTKRTCPRGYEYVSCWWGATCTYGGRCSGSRDDGSLTYEFHVDA |
| TTVHQETKKSCPEGYTYVWGCDDDSGGVGYGCAPNGASSCSFTYTYEFHIDA |
| TAVHQRTKRTCPEGLVYNSDQSRCCAADSGVCWEYWRGERVTRGFTYEWYVEA |
| SIVHQRTQTSKGCPEGWNDCGGNTYGYDCGRWGCGHYLNSGPRISAYQTTYNYEWYVDA |
| SIVHQKTQTSEGCPEGWSECGVGTYGYDCGRWGCGHYLNTGPLISGYVTTNKYEWHVEA |
| STVHQKAHTSVACPEGWSECGVAIYGYDCGRWGCGHFLNSGPNISPYVTTDAYEWYVDA |
| SIVHQKAHTSVTCPEGWSECGVAIYGYECGRWGCGHFLNSGPNISPYVSTHKYEWYVDA |
| STVHQKAHTSVACPEGWSECGVAIYGYDCGRWGCGHFLNSGPNISPYVSTHKYEWYVDA |
| TTVHQKAHTSVACPEGWSECGVAIYGYDCGRWGCGHFLNSGPNISPYVTTDAYEWYVDA |
| TTIQQLTERTCPEGSMLGSECNSHWSCEGCDCAKHCTWGGRCVDCSPYMSTHEWHIET |
| TSVYQKTDTIRHPCRDDSSYACVCRWTRGCSGTDCSGCTPDSDIDYGCDTIACNYTYQLYVDA |
| TTVHQHSNNKKTCPDGTSSHSACILGTGGCCLDQYYRRGICGRVDACYEYSSSVNYEWYVDA |
| ATVHQRTERSCPDGSSDAESGVCSGCCRGWDCCSFEVDWVGCKGCTAYTYRTVYEHHVDA |
| TTVHQQTKTKKNPCRDVASPVCVCRWAEGCSGTDCSECTPDPDRDYGTCEIIACTHTYELHVDA |
| TTVIQKTATKQSCPDDYRDGGECCIYGRCSAEDCSVTGWEYYGSTLCRVPYITTHAYQWHVDA |
| TTVHQETRRNCPDGYSEINACGDRYKASGGLCCGEGAGAWRCWECSDTIIPTTTYEFYVDA |
| TTVHQETRRHCPDGYSDIYGCGHYYSATGGHCCGEGAGAWRCWECSDTIMPSTTYEFYVDA |
| TTVHQETRRNCPDGYSDIYGCGNRYAATGGHCCGEGAGAWRCWECSDSIWPSSTYEFYVDA |
| VIVYQETIKSCREGYIDGGGCCLPGSCRGCACSYYDWLKCPRDCRGTSEEYIYTYNFRVDA |
| STVHQLTITTLGCPDGVSVVNTCGWLRCNCGDSIYCSRSADSGMWCGRCGDCTSTHTHQWHVDA |
| STVHQLTITTLGCPDGVSVVPTCGWLRCNCGEDLYCSRSDEQGTWCGRCGDCTSTYTHQWHVDA |
| STVHQLTITTVGCPNGVTRVATCGWKRCHCGENIYCSRSDDSGTWCGRCGDCTGTYTYQWHVDA |
| TTVHQKTIAKCPDGYTYSGDCGICDDCGGRTSRAYDCAGDTSLYMCGRRSPTLLTYQFHVDV |
| ATVHQQTKKQTERSCPDGYTYINDCIGASGAVSRYDCWRFRRMNGVCIDGTYSTTADTYTYEFHVDA |
| TTVHQKTRKSCPGGCRDTDGHDYDHWSCAGSDCCCFGTDGGCGRWGIYCSHSYTYTYEYHVET |
| CTVQQKTHQVCPDGFNWGYGCAAGSSRFCTRHDWCCYDERADSHTYGFCTGNRVTNTYEFHADA |
| TIVHQKTKREERCPAGYSISACRDGIGCGATDCCADGATDYAWGWECKSRIYGDSYEFHVDA |
| GIVVQRTYERRTCPDTFTYKDGCRRGGTLLNSRSGCYNVYCNYHDAEVTYAHRWYVDA |
| TTVHQRTKKTCPLGYDLNDRCDHFNTCRVEECCKNGVVNAYGICEYAGGSATYTYEWYVDA |
| TIVHQETNKEKICRVDYVDSATCTWNCDCCRSRKSDCCAYANSRSCWNTSGTYTYTYEFHVDA |
| TTVHQTTNRKKTCPDNYREVDGCDPYDCCLTTWCTNSYCTRYIYEDSYEFYVTA |
| TSVLQSTKKQKSCPDGLSYRAWDDFCCPNVGRCLPPINTYTYTHAFHIEA |
| TTVHQKTITSCPDGYVYSYDCGICDDCGGRTSRAYDCAGDTSLYMCGRRSPSSAYQFHVDR |
| TTVYQNTRSKERSCPYGTGFDPTWCDSVLPCRRDGCWTTVWGCCEGDVDGGETTPTYEFYVDA |
| ATVHQKTTEKKTCPDGGEPSVICLDASEVCRISECYEDHPTTIYTYTYEFHVDA |
| TTVHQRTIKSGCPPGYKSGVDCSPGSECKWGCYAVDGRRYGGYGADSGVGSTYTHEFYVDA |
| TTVYQKTRSDCPAGYKQVYGCSAGNCGCRGNGCCNSGSCGTWSEWGQYGCCNCHSSYEFHVDA |
| TSVYQKTTKRFTCHDPSGGTWERADGATSCPGTHCCSYGRDGIWHGYDRRRTYTEVFTYELDVEE |
| TTVHQKTKKSCPLGYAINDRCDDLKTCGPDECCLNGVVNAYGICEYEGESATHTYEWYVDA |
| CTVYQKTETKKSCPDGYRFFQECRGTGTGCPGDDCVCYDGRGGFRWRNGCTTYTYTYRHNLHVET |
| STVYQETKRKCPDGYRVGTDCTPGKGCDYACHSRLGVRWGGDGRDGGRGYIVSYELHIDA |
| TTVYQKTKETCPDGYIWAERCPGGWTSCRNACWLEGGDSAGAYDEVTSTVHRYEFYVDT |
| TTVHQRTITRCPDDFGNTCRCSKGTCPCGEDACCGTNQYSFWGDCRDVGRTTFIETYEWNVDD |
| GAVYQKTNEQSSCPDGWRDTGTHCEDYGSWGYRDYTFTYTYEFHVHN |
| TTVHQTTRKTQSCPDGYTDIDGCSWRHGCCRYDCCSDRSCSWCVDRDWSSYIVTATYELDIEA |
| VTVQQQTKLEYSCPNGYSSDAGCLAAWRCGDYDCCRENAFRPCTGSIPTSNYEWHLEA |
| TTLYQNTRKKGGCPEGTTYLGGSSETYRCGLEGRMRTYSYTYSYEWYVDA |
| ITAHQKTNKIPHCRDGYDYGGGCCVSSGVYGESCRSSGGSDCDQWVGCESVTYTETYEWHVDA |
| TAVHQQTERSCPPDTTEHDCCGCGGRGCAWSGCYRKGYGTGCRVCTSIQARDYIYTYKLHIDT |
| TTVYQSTRKTSRNCPDGGSPSVQCLDDTWACRIVDCYDDGTYGTYRFTNTYDWYVDA |
| TTVHQKTDQKRSSCPDGYSDCLVCGADRDGCSSGGCRGCWTNAYYSSRTYYNTDEFHYKPNEFHVDM |
