# Supplementary material for: Isolation of antigen-specific, disulphide-rich knob domain peptides from bovine antibodies
Source: PLoS Biol. 2020 Sep 4;18(9):e3000821. doi: 10.1371/journal.pbio.3000821 (PMC7498065; doi:10.1371/journal.pbio.3000821)
Supplement: S3 Table — Sequences chosen for reformatting as PGT121-knob domain fusion proteins are shown in bold. (DOCX) [file pbio.3000821.s012.docx]

| **Ultralong CDR-H3 Sequence** |
| --- |
| **TSVLQSTKPQKSCPDGFSYRSWDDFCCPMVGRCLAPRNTYTTEFTIEA** |
| STVHQKAHTSVACPEGWSECGVAIYGYDCGRWGCGHFLNSGPNISPYVSTHKYEWYVDA |
| TTVHQKAHTSVACPEGWSECGVAIYGYDCGRWGCGHFLNSGPNISPYVTTDAYEWYVDA |
| SIVHQKTQTSEGCPEGWSECGVGTYGYDCGRWGCGHYLNTGPLISGYVTTNKYEWHVEA |
| STVHQKAHTSVACPEGWSECGVAIYGYDCGRWGCGHFLNSGPNISPYVTTDAYEWYVDA |
| **SIVHQKAHTSVTCPEGWSECGVAIYGYECGRWGCGHFLNSGPNISPYVSTHKYEWYVDA** |
| **VIVYQETIKSCREGYIDGGGCCLPGSCRGCACSYYDWLKCPRDCRGTSEEYIYTYNFRVDA** |
| **CTVQQKTHQVCPDGFNWGYGCAAGSSRFCTRHDWCCYDERADSHTYGFCTGNRVTNTYEFHADA** |
| TTVHQRTITRCPDDFGNTCRCSKGTCPCGEDACCGTNQYSFWGDCRDVGRTTFIETYEWNVDD |
| CTTVHQKTDQKRSSCPDGYSDCLVCGADRDGCSSGGCRGCWTNAYYSSRTYYNTDEFHYKPNEFHVDM |
| **TTVHQRTIKSGCPPGYKSGVDCSPGSECKWGCYAVDGRRYGGYGADSGVGSTYTHEFYVDA** |
| **TTVHQTTNRKKTCPDNYREVDGCDPYDCCLTTWCTNSYCTRYIYEDSYEFYVTA** |
| TTVHQKTKKSCPLGYAINDRCDDLKTCGPDECCLNGVVNAYGICEYEGESATHTYEWYVDA |
| TSVLQSTKKQKSCPDGLSYRAWDDFCCPNVGRCLPPINTYTYTHAFHIEA |
