# Supplementary material for: Isolation of antigen-specific, disulphide-rich knob domain peptides from bovine antibodies
Source: PLoS Biol. 2020 Sep 4;18(9):e3000821. doi: 10.1371/journal.pbio.3000821 (PMC7498065; doi:10.1371/journal.pbio.3000821)
Supplement: S4 Table — Summary of kinetics from n = 3. (for individual occasions see S4B Table, S4C Table and S4D Table). (DOCX) [file pbio.3000821.s013.docx]

**S4A Table. Biacore single-cycle kinetics data on PGT121 Fab – knob domain fusion proteins.**

Summary of kinetics from *n=3*. (for individual occasions see S8B Table, S8C Table and S8D Table).

|  | **Mean k_on_ (1/Ms)** | **Mean k_off_ (1/s)** | **Mean KD (M)** | **Mean Stoichiometric**  **ratio at 1 µM** |
| --- | --- | --- | --- | --- |
| **PGT-121 K8** | 7.82E+03 | 2.04E-05 | 2.68E-09 | 0.3 |
| **PGT-121 K57** | 4.21E+04 | 1.41E-04 | 3.36E-09 | 0.9 |
| **PGT-121 K60** | **-** | - | - | <0.1 |
| **PGT-121 K92** | 3.59E+04 | <1.18E-05^a^ | <3.00E-10^a^ | 0.9 |
| **PGT-121 K136** | 4.76E+03 | <1.00E-05^a^ | <2.19E-09^a^ | 0.8 |
| **PGT-121 K149** | 3.75E+03 | 1.64E-03 | 5.17E-07 | 0.5 |

**S4B Table. Biacore single-cycle kinetics data on PGT121 Fab – knob domain fusion proteins, *n=1***

|  | **Kinetics Chi² (RU²)** | **k_on_ (1/Ms)** | **k_off_ (1/s)** | **KD (M)** | **Rmax (RU)** | **tc** | **Stoichiometric ratio at 1 µM** |
| --- | --- | --- | --- | --- | --- | --- | --- |
| **PGT-121 K8** | 0.092 | 8.05E+03 | 2.25E-05 | 2.8E-09 | 14.6 | 4.27E+10 | 0.30 |
| **PGT-121 K57** | 0.481 | 3.95E+04 | 1.53E-04 | 3.88E-09 | 43.6 | 3.00E+12 | 0.89 |
| **PGT-121 K60** | - | - | - | - | - | - | <0.1 |
| **PGT-121 K92** | 1.143 | 3.96E+04 | <1.0E-05^a^ | <2.53E-10^a^ | 44.8 | 7.12E+10 | 0.91 |
| **PGT-121 K136** | 0.282 | 5.48E+03 | <1.0E-05^a^ | <1.82E-09^a^ | 38.9 | 6.88E+10 | 0.82 |
| **PGT-121 K149** | 0.13 | 5.27E+03 | 1.49E-03 | 2.83E-07 | 13.00 | 3.58E+10 | 0.5 |

**S4C Table. Biacore single-cycle kinetics data on PGT121 Fab – knob domain fusion proteins, *n=2***

|  | **Kinetics Chi² (RU²)** | **k_on_ (1/Ms)** | **k_off_ (1/s)** | **KD (M)** | **Rmax (RU)** | **tc** | **Stoichiometric ratio at 1 µM** |
| --- | --- | --- | --- | --- | --- | --- | --- |
| **PGT-121 K8** | 0.12 | 6.61E+03 | 2.25E-05 | 3.41E-09 | 12.6 | 1.33E+10 | 0.30 |
| **PGT-121 K57** | 1.95 | 4.47E+04 | 1.24E-04 | 2.77E-09 | 41.3 | 5.2E+10 | 0.9 |
| **PGT-121 K60** | - | - | - | - | - | - | <0.1 |
| **PGT-121 K92** | 5.25 | 3.88E+04 | 1.18E-05 | 3.04E-10 | 42.2 | 9.6E+11 | 0.9 |
| **PGT-121 K136** | 2.31 | 3.52E+03 | <1.0E-05^a^ | <2.84E-09^a^ | 38.4 | 2.3E+10 | 0.8 |
| **PGT-121 K149** | 0.12 | 3.84E+03 | 1.62E-03 | 4.25E-07 | 10.00 | 3.29E+09 | 0.5 |

**S4D Table. Biacore single-cycle kinetics data on PGT121 Fab – knob domain fusion proteins, *n=3***

|  | **Kinetics Chi² (RU²)** | **k_on_ (1/Ms)** | **k_off_ (1/s)** | **KD (M)** | **Rmax (RU)** | **tc** | **Stoichiometric ratio at 1 µM** |
| --- | --- | --- | --- | --- | --- | --- | --- |
| **PGT-121 K8** | 9.10E-02 | 8.81E+03 | 1.61E-05 | 1.82E-09 | 14.2 | 4.77E+10 | 0.3 |
| **PGT-121 K57** | 4.16E-01 | 4.22E+04 | 1.45E-04 | 3.43E-09 | 39.7 | 9.42E+11 | 0.9 |
| **PGT-121 K60** | - | - | - | - | - | - | <0.1 |
| **PGT-121 K92** | 2.92E+00 | 2.92E+04 | <1.0E-05^a^ | <1.42E-10^a^ | 51.4 | 2.68E+10 | 1.0 |
| **PGT-121 K136** | 1.15E+00 | 5.28E+03 | <1.0E-05^a^ | <1.89E-09^a^ | 40.4 | 1.00E+08 | 0.9 |
| **PGT-121 K149** | 0.15 | 2.15E+03 | 1.81E-03 | 8.42E-07 | 10.00 | 1.18E+10 | 0.4 |

^a^ K_off_ capped at 1.00E-05 s^-1^ as the measured K_off_ exceeded limit of determination by Biacore (<1.00E-05 s^-1^). K_D_ values may be under-estimates of affinity
