# Supplementary material for: Isolation of antigen-specific, disulphide-rich knob domain peptides from bovine antibodies
Source: PLoS Biol. 2020 Sep 4;18(9):e3000821. doi: 10.1371/journal.pbio.3000821 (PMC7498065; doi:10.1371/journal.pbio.3000821)
Supplement: S5 Table — (DOCX) [file pbio.3000821.s014.docx]

| **ID** | **Knob domain peptide sequence** |
| --- | --- |
| K8 | GVCPDGFNWGYGCAAGSSRFCTRHDWCCYDERADSHTYGFCTGNRVENLYFQ |
| K57 | GSGCPPGYKSGVDCSPGSECKWGCYAVDGRRYGGYGADSGVENLYFQ |
| K60 | GKSCREGYIDGGGCCLPGSCRGCACSYYDWLKCPRDCRGTSEEENLYFQ |
| K92 | GVTCPEGWSECGVAIYGYECGRWGCGHFLNSGPNISPYVTTGSENLYFQ |
| K136 | GTCPDNYREVDGCDPYDCCLTTWCTNSYCTRYIENLYFQ |
| K149 | GSCPDGFSYRSWDDFCCPMVGRCLAPRNGSENLYFQ |
