# Supplementary material for: Isolation of antigen-specific, disulphide-rich knob domain peptides from bovine antibodies
Source: PLoS Biol. 2020 Sep 4;18(9):e3000821. doi: 10.1371/journal.pbio.3000821 (PMC7498065; doi:10.1371/journal.pbio.3000821)
Supplement: S6 Table — Data are shown for the 4+ charge state. DSB, disulphide bonds (DOCX) [file pbio.3000821.s015.docx]

|  | **Molecular Formula** | **#DSB** | **Formula**  **(with DSBs)** | **(M+4H)^4+^** | **Predicted Monoisotopic mass (with DSBs)** | **Observed mean Monoisotopic mass** | **Δm** |
| --- | --- | --- | --- | --- | --- | --- | --- |
| **K8** | C_259_H_359_N_75_O_81_S_6_ | 3 | C_259_H_353_N_75_O_81_S_6_ | C_259_H_357_N_75_O_81_S_6_ | 6001.413 Da | 6001.361 Da | -8.7 ppm |
| **K57** | C_214_H_309_N_59_O_72_S_4_ | 2 | C_214_H_305_N_59_O_72_S_4_ | C_214_H_309_N_59_O_72_S_4_ | 4981.089 Da | 4981.071 Da | -3.5 ppm |
| **K60** | C_229_H_345_N_67_O_77_S_8_ | 4 | C_229_H_337_N_67_O_77_S_8_ | C_229_H_341_N_67_O_77_S_8_ | 5513.227 Da | 5513.223 Da | -0.9 ppm |
| **K92** | C_244_H_347_N_63_O_78_S_4_ | 2 | C_244_H_343_N_63_O_78_S_4_ | C_244_H_347_N_63_O_78_S_4_ | 5534.373 Da | 5534.400 Da | 4.9 ppm |
| **K136** | C_201_H_287_N_51_O_69_S_6_ | 3 | C_201_H_281_N_51_O_69_S_6_ | C_201_H_285_N_51_O_69_S_6_ | 4704.833 Da | 4704.812 Da | -4.5 ppm |
| **K149** | C_181_H_261_N_51_O_57_S_5_ | 2 | C_181_H_257_N_51_O_57_S_5_ | C_181_H_261_N_51_O_57_S_5_ | 4221.74‬0 Da |  | 8.5 ppm |
