# Supplementary material for: Isolation of antigen-specific, disulphide-rich knob domain peptides from bovine antibodies
Source: PLoS Biol. 2020 Sep 4;18(9):e3000821. doi: 10.1371/journal.pbio.3000821 (PMC7498065; doi:10.1371/journal.pbio.3000821)
Supplement: S7 Table — Summary of kinetics from n = 3 occasions (for individual occasions see S7B Table, S7C Table and S7D Table). (DOCX) [file pbio.3000821.s016.docx]

**S7A Table. Biacore single-cycle kinetics data on isolated knob domain peptides.**

Summary of kinetics from *n=3* occasions (for individual occasions see S7B Table, S7C Table and S7D Table).

|  | **Mean k_on_ (1/Ms)** | **Mean k_off_ (1/s)** | **Mean KD (M)** | **Mean Stoichiometric**  **ratio at 1 µM** |
| --- | --- | --- | --- | --- |
| **K8** | 2.61E+04 | 3.57E-04 | 1.73E-08 | 0.6 |
| **K57** | 2.98E+05 | 4.08E-04 | 1.40E-09 | 0.7 |
| **K60** | **-** | - | - | <0.1 |
| **K92** | 1.24E+05 | <1.13E-04 | <6.03E-10 | 0.8 |
| **K136** | 1.94E+04 | 1.23E-04 | 6.40E-09 | 0.5 |
| **K149** | 2.38E+05 | 3.68E-03 | 1.55E-08 | 1.0 |

**S7B Table. Biacore single-cycle kinetics data on isolated knob domain peptides, *n=1***

|  | **Kinetics Chi²**  **(RU²)** | | **k_on_ (1/Ms)** | **k_off_ (1/s)** | **K_D_ (M)** | **Rmax (RU)** | **tc** | **stoichiometric ratio at 1 µM** |
| --- | --- | --- | --- | --- | --- | --- | --- | --- |
| **K8** | | 19.1 | 8.56E+03 | 2.28E-04 | 2.66E-08 | 48.7 | 1.4E+12 | 0.9 |
| **K57** | | 1.4 | 2.58E+05 | 3.85E-04 | 1.49E-09 | 49.2 | 4.5E+11 | 0.8 |
| **K60** | | - | - | - | - | - | - | <0.1 |
| **K92** | | 22.0 | 8.40E+04 | <1.0E-05 | <1.1E-10 | 62.7 | 1.1E+11 | 1.0 |
| **K136** | | 2.9 | 1.52E+04 | 1.11E-04 | 7.25E-09 | 42.1 | 1.2E+10 | 0.6 |
| **K149** | | 0.72 | 2.38E+05 | 3.68E-03 | 1.55E-08 | 11.5 | 8.51E+13 | 1.0 |

**S7C Table. Biacore single-cycle kinetics data on isolated knob domain peptides, *n=2***

|  | **Kinetics Chi²**  **(RU²)** | | **k_on_ (1/Ms)** | **k_off_ (1/s)** | **K_D_ (M)** | **Rmax (RU)** | **tc** | **stoichiometric ratio at 1 µM** |
| --- | --- | --- | --- | --- | --- | --- | --- | --- |
| **K8** | | 1.36 | 2.70E+04 | 4.05E-04 | 1.50E-08 | 20.3 | 8.88E+08 | 0.4 |
| **K57** | | 0.22 | 2.48E+05 | 3.05E-04 | 1.23E-09 | 19.6 | 6.19E+13 | 0.7 |
| **K60** | | - | - | - | - | - | - | <0.1 |
| **K92** | | 0.95 | 1.16E+05 | 1.37E-04 | 1.18E-09 | 35.2 | 1.14E+11 | 0.7 |
| **K136** | | 0.42 | 2.20E+04 | 1.72E-04 | 7.85E-09 | 20.7 | 6.93E+10 | 0.4 |
| **K149** | | 0.21 | 1.70E+05 | 4.53E-03 | 2.67E-08 | 10.3 | 6.51E+10 | 0.9 |

**S7D Table. Biacore single-cycle kinetics data on isolated knob domain peptides, *n=3***

|  | **Kinetics Chi²**  **(RU²)** | | **k_on_ (1/Ms)** | **k_off_ (1/s)** | **K_D_ (M)** | **Rmax (RU)** | **tc** | **stoichiometric ratio at 1 µM** |
| --- | --- | --- | --- | --- | --- | --- | --- | --- |
| **K8** | | 1.3 | 4.28E+04 | 4.38E-04 | 1.02E-08 | 27.8 | - | 0.5 |
| **K57** | | 1.9 | 3.49E+05 | 3.41E-04 | 9.76E-10 | 41.6 | 1.45E+13 | 0.7 |
| **K60** | | - | - | - | - | - | - | <0.1 |
| **K92** | | 3.4 | 1.73E+05 | 8.83E-05 | 5.10E-10 | 40.3 | 1.68E+12 | 0.8 |
| **K136** | | 4.3 | 2.10E+04 | 8.59E-05 | 4.09E-09 | 32.0 | 1.65E+12 | 0.6 |
| **K149** | | 1.08 | 3.35E+05 | 4.04E-03 | 1.21E-08 | 11.5 | 3.63E+14 | 1.0 |

^a^ K_off_ capped at 1.00E-05 s^-1^ as the measured K_off_ exceeded limit of determination by Biacore (<1.00E-05 s^-1^). K_D_ values may be under-estimates of affinity.
