# Supplementary material for: Isolation of antigen-specific, disulphide-rich knob domain peptides from bovine antibodies
Source: PLoS Biol. 2020 Sep 4;18(9):e3000821. doi: 10.1371/journal.pbio.3000821 (PMC7498065; doi:10.1371/journal.pbio.3000821)
Supplement: S8 Table — Summary data table for n = 3 experiments. For 2-hour data, refer to S8A Table and for 24-hour data, refer to S8B Table. (DOCX) [file pbio.3000821.s017.docx]

**S8A Table.** **FRET assay K_D_ *app* for PGT121 fusion proteins binding to C5-Tb after a 2 hour incubation.**

Summary data table for n=3 experiments.

|  | ***n1*** | ***n2*** | ***n3*** | **Geomean K_D_ *app* (nM)** |
| --- | --- | --- | --- | --- |
| **PGT121 K8** | 6.3 | 17.9 | 4.7 | 8.1 |
| **PGT121 K57** | 4.9 | 3.0 | 3.4 | 3.7 |
| **PGT121 K92** | 14.0 | 10.3 | 15.5 | 13.1 |
| **PGT121 K136** | NR | 46.3 | 115.0 | 73.0 |
| **PGT121 K149** | 34.9 | 57.6 | 30.1 | 39.3 |

**S8B Table. FRET assay K_D_ *app* for PGT121 fusion proteins binding to C5-Tb after a 24 hour incubation.**

Summary data table for n=3 experiments.

|  | ***n1*** | ***n2*** | ***n3*** | **Geomean K_D_ *app* (nM)** |
| --- | --- | --- | --- | --- |
| **PGT121 K8** | 6.6 | 5.0 | 4.7 | 5.4 |
| **PGT121 K57** | 3.3 | 2.3 | 3.4 | 3.0 |
| **PGT121 K92** | 19.3 | 7.7 | 11.3 | 11.9 |
| **PGT121 K136** | 46.3 | 23.4 | 58.1 | 39.8 |
| **PGT121 K149** | 126.6 | 90.6 | 24.9 | 65.9 |
