# Supplementary material for: Isolation of antigen-specific, disulphide-rich knob domain peptides from bovine antibodies
Source: PLoS Biol. 2020 Sep 4;18(9):e3000821. doi: 10.1371/journal.pbio.3000821 (PMC7498065; doi:10.1371/journal.pbio.3000821)
Supplement: S9 Table — Summary data table for n = 3 experiments. (DOCX) [file pbio.3000821.s018.docx]

**S9 Table. Competition FRET assays to derive IC50 and Ki values for knob domain peptides.**

Summary data table for n=3 experiments.

|  | ***n1*** | ***n2*** | ***n3*** | **Geomean IC50 (nM)** | **Ki (nM)** |
| --- | --- | --- | --- | --- | --- |
| K8 | 20.7 | 31.2 | 26.1 | 25.6 | 12.8 |
| K57 | 4.0 | 3.2 | 3.5 | 3.6 | 1.8 |
| K92 | 7.6 | 7.4 | 7.3 | 7.4 | 3.7 |
| K136 | 44.9 | 31.2 | 38.8 | 37.9 | 18.9 |
| K149 | 54.3 | 45.5 | 61.7 | 53.4 | 26.7 |
